# Vertical Redistribution of Soil Organic Carbon Pools After Twenty Years of Nitrogen Addition in Two Temperate Coniferous Forests

**DOI:** 10.1007/s10021-018-0275-8

**Published:** 2018-06-26

**Authors:** Stefan J. Forstner, Viktoria Wechselberger, Stefanie Müller, Katharina M. Keibinger, Eugenio Díaz-Pinés, Wolfgang Wanek, Patrick Scheppi, Frank Hagedorn, Per Gundersen, Michael Tatzber, Martin H. Gerzabek, Sophie Zechmeister-Boltenstern

**Affiliations:** 1 Institute of Soil Research, Department of Forest and Soil Sciences, University of Natural Resources and Life Sciences (BOKU), 1190 Vienna, Austria; 2 Department of Microbiology and Ecosystem Science, University of Vienna, 1090 Vienna, Austria; 3 Swiss Federal Institute for Forest, Snow and Landscape Research (WSL), 8903 Birmensdorf, Switzerland; 4 Department of Geosciences and Natural Resource Management, University of Copenhagen, 1958 Frederiksberg C, Denmark; 5 Division of Radiation Protection, Department of Radiation Protection and Radiochemistry, Austrian Agency for Health and Food Safety (AGES), 1220 Vienna, Austria

**Keywords:** carbon sequestration, carbon mineralization, nitrogen deposition, nitrogen saturation, fine roots, exchangeable cations, soil carbon, soil pH, Norway spruce

## Abstract

Nitrogen (N) inputs from atmospheric deposition can increase soil organic carbon (SOC) storage in temperate and boreal forests, thereby mitigating the adverse effects of anthropogenic CO_2_ emissions on global climate. However, direct evidence of N-induced SOC sequestration from low-dose, long-term N addition experiments (that is, addition of < 50 kg N ha^−1^ y^−1^ for > 10 years) is scarce worldwide and virtually absent for European temperate forests. Here, we examine how tree growth, fine roots, physicochemical soil properties as well as pools of SOC and soil total N responded to 20 years of regular, low-dose N addition in two European coniferous forests in Switzerland and Denmark. At the Swiss site, the addition of 22 kg N ha^−1^ y^−1^ (or 1.3 times throughfall deposition) stimulated tree growth, but decreased soil pH and exchangeable calcium. At the Danish site, the addition of 35 kg N ha^−1^ y^−1^ (1.5 times throughfall deposition) impaired tree growth, increased fine root biomass and led to an accumulation of N in several belowground pools. At both sites, elevated N inputs increased SOC pools in the moderately decomposed organic horizons, but decreased them in the mineral topsoil. Hence, long-term N addition led to a vertical redistribution of SOC pools, whereas overall SOC storage within 30 cm depth was unaffected. Our results imply that an N-induced shift of SOC from older, mineral-associated pools to younger, unprotected pools might foster the vulnerability of SOC in temperate coniferous forest soils.

**Electronic supplementary material:**

The online version of this article (10.1007/s10021-018-0275-8) contains supplementary material, which is available to authorized users.

## Introduction

Reactive nitrogen (N_*R*_) inputs from atmospheric deposition to terrestrial ecosystems have more than doubled since the onset of the industrial revolution, mainly due to agricultural intensification and fossil fuel burning (Galloway and others 2008). Further increases in global N_*R*_ deposition rates are predicted up to 2050 (Galloway and others 2004; Simpson and others 2014). Temperate forests, which covered 7.67 × 10^6^ km^2^ globally in 2007 (Pan and others 2011), are particularly affected by high N_*R*_ deposition in the vicinity of the densely populated areas in North America, Europe and Asia (Townsend and others 1996; Holland and others 1997). At the same time, these forests were a net sink for atmospheric CO_2_ of up to 0.8 Pg of carbon (C) per year during the early 2000s (Pan and others 2011) corresponding to about 1/3 of the ‘residual land sink’ (Ciais and others 2014).

The net uptake of C by temperate forests has been linked to anthropogenic N_*R*_ inputs (Oren and others 2001; Reay and others 2008; Fernández-Martínez and others 2014), which help to alleviate N limitation of tree growth (Tamm 1991; LeBauer and Treseder 2008; Thomas and others 2010). Hence, most of the additionally sequestered C is stored in tree biomass (Pregitzer and others 2008; De Vries and others 2009; Frey and others 2014). However, the vegetation contains only 40% of C stored in temperate forest ecosystems, while soils down to 1 m depth account for the remaining 60%, mainly in the form of soil organic carbon (SOC; Dixon and others 1994; Lal 2005). It is therefore vital to quantify N-induced changes in SOC in order to assess the overall effects of N_*R*_ deposition on temperate forest C (De Vries and others 2006, 2014).

Several meta-analyses suggest that experimental N addition increases the amount of SOC in temperate forests (Hyvönen and others 2008; Nave and others 2009; Janssens and others 2010; Liu and Greaver 2010; Yue and others 2016), although this response is not universal (Lu and others 2011b). The main mechanisms behind the observed increases in SOC appear to be higher litter inputs to soil via stimulated tree productivity and/or suppressed decomposition of recalcitrant soil organic matter (SOM; Franklin and others 2003; Janssens and others 2010; Hagedorn and others 2012). However, amount and location of the additional C sequestered in SOM may vary considerably between ecosystems and soil horizons (Nave and others 2009; Liu and Greaver 2010; Yue and others 2016). Part of these inconsistencies might arise from the fact that few studies evaluated changes in SOC pools, as opposed to SOC concentrations, in experiments explicitly simulating the long-term effects of low-dose N addition (for example, Pregitzer and others 2008). In such experiments, it is crucial to distinguish between N-induced changes in SOC concentrations and SOC pools, respectively, as only the latter represent the actual amount of C stored in a defined soil volume/area. This is even more important as N has been shown to affect concentrations and pools of SOC differently (Maaroufi and others 2015; Boot and others 2016). We are aware of only two studies that quantified SOC pools in temperate forests after more than 10 years of experimental N addition. Pregitzer and colleagues (2008) found that SOC pools in the top 10 cm of mineral soil in a hardwood forest increased by 25% on average after 10 years of adding N at 30 kg ha^−1^ y^−1^. More recently, Frey and colleagues (2014) reported that organic horizon SOC pools increased by 33 and 52% in hardwood and pine stands, respectively, whereas in mineral horizons SOC pools did not respond to 20 years of N addition treatment (50 kg ha^−1^ y^−1^).

Fine roots represent a relatively small fraction of total plant biomass in temperate forests (4–7%; Vogt and others 1995). However, fine root production is a large component of belowground net primary production (BNPP) in these systems (Nadelhoffer 2000), which in turn can comprise up to 45% of the total belowground C flux (TBCF; Litton and Giardina 2008). Furthermore, root-derived C has increasingly been recognized as an important contributor to SOC (Rasse and others 2005; Crow and others 2009; Tefs and Gleixner 2012; Angst and others 2018). Thus, even small changes in fine root biomass (FRB) in response to altered N inputs may feed back on ecosystem C storage (Xia and Wan 2008; Janssens and others 2010; Li and others 2015; Peng and others 2017).

Typically, FRB decreases with increasing N availability, especially relative to aboveground biomass (Nadelhoffer and others 1985; Gundersen and others 1998; Yuan and Chen 2010). A recent review found a 13.5% reduction of FRB in forests exposed to experimentally increased N deposition for up to 14 years (Li and others 2015). However, results from the few long-term experiments (≥ 15 years of N addition) are more ambiguous: While higher N inputs reduced FRB in a temperate coniferous forest stand (Frey and others 2014), FRB was not affected by enhanced N inputs in a temperate deciduous (Burton and others 2012; Frey and others 2014) or in a boreal coniferous forest (Maaroufi and others 2015).

Here, we studied two temperate coniferous forests located in Switzerland and Denmark, which have received low doses of additional N in monthly or more frequent intervals for two decades. We quantified the amount of C and N stored in soil and fine root pools after 19 and 20 years of treatment, respectively, to test if experimental N addition increased SOC and soil total N (STN) pools (Hypothesis 1) and decreased fine root C (and N) pools (Hypothesis 2). In addition, we combine measurements of soil pH, exchangeable cations and extractable N pools with long-term monitoring data on tree growth to assess the effects of N addition on an ecosystem level.

## Materials and Methods

### Study Sites

We studied two temperate coniferous forest sites at Alptal (Switzerland) and Klosterhede (Denmark), which have received low-dose N addition for approximately two decades (see Table 1 for site characteristics). Alptal is located in a valley on the northern edge of the Alps in central Switzerland (47°02′N, 8°43′E). The local climate is wet and cool with a mean annual precipitation of 2300 mm and a mean annual temperature of 6°C. The experimental site is located on a 20% slope with a western aspect. The soils are clay-rich Gleysols that have developed from Flysch, a sedimentary rock formation consisting of alternating calcareous sandstones with argillite and bentonite schists (Leupold 1942; Hagedorn and others 2001b). The heterogeneous microtopography of mounds and depressions results in a patchy distribution of soil types (Schleppi and others 1998, 2017; Hagedorn and others 2001b; Krause and others 2013). On mounds, Umbric Gleysols dominate with a thick organic layer (Oi, Oe and Oa horizons) on top of an Ah, an oxidized Bl and a partly oxidized Blr horizon. In depressions, the organic layer consists of an Oi horizon, whereas Oe and Oa horizons are mostly lacking, so that the soil classifies as Mollic Gleysol (Schleppi and others 1998; Hagedorn and others 2001b; Krause and others 2013). Ground vegetation is well developed, and different botanical associations are found on mounds and in depressions, respectively (Schleppi and others 1999b). The naturally regenerating forest stand is dominated by up to 260-year-old Norway spruce trees (*Picea abies* L. Karst.) with a 15% contribution of silver fir (*Abies alba* Mill.). Bulk N deposition from the atmosphere consists equally of ammonium (NH_4_^+^) and nitrate (NO_3_^−^) and approximates to 12 kg N ha^−1^ y^−1^ (Schleppi and others 1999a).

**Table 1:** Selected Site Characteristics of Alptal (CH) and Klosterhede (DK)

	Alptal	Klosterhede
Latitude	47°02′N	56°29′N
Longitude	8°43′E	8°24′E
MAT (°C)	6	9
MAP (mm)	2300	860
Elevation (masl)	1200	27
Slope (%)	20	0
Dominant tree species	*Picea abies* (L.) Karst.	*Picea abies* (L.) Karst.
Stand age (years)	Up to 260	97
Density of stems > 10 cm DBH (ha^−1^)	430	860
Dominant tree height (m)	30	20
Basal area (m^2^ ha^−1^)	41	30
Microtopography	Mounds, depressions	Homogenous
Soil type	Umbric/Mollic Gleysols	Haplic Podzol
Soil horizon sequence	Oi/Oe/Oa/Ah/Bl/Blr	(Oi^a^)/Oe/Oa/AE/E/Bh/Bs
Parent material	Flysch	Glacio-fluvial sands
Bulk N deposition (kg N ha^−1^ y^−1^)	12.3^b^	9.4^c^
Throughfall N deposition (kg N ha^−1^ y^−1^)	16.8^b^	23.0^c^
Experimental N addition (kg N ha^−1^ y^−1^)	22	35
Form of added N	NH_4_NO_3_	NH_4_NO_3_
Frequency of N addition	In each rain event	Monthly
Start (duration) of N addition	1995 (19 years)	1992 (20 years)^d^

Data for Alptal were compiled from Schleppi and others (1998), Hagedorn and others (2001a, b) and Krause and others (2012a, b, 2013). Data for Klosterhede were compiled from Gundersen and Rasmussen (1995) and Gundersen (1998).

^a^The Oi horizon is not included in this study.

^b^Average of two experimental catchments from April 1993 to March 1995.

^c^Average of control plots from 1988 to 1992.

^d^No treatment in 1998–1999.

The Klosterhede experimental site is located in Western Jutland, Denmark (56°29′N, 8°24′E), with a mean annual temperature of 9°C and a mean annual precipitation of 860 mm. The site is flat and microtopography is more uniform compared to Alptal. The coarse-textured, nutrient-poor soil is classified as Haplic Podzol and has developed from glacio-fluvial sands (Gundersen and Rasmussen 1995). A thick organic layer consisting of Oi (not sampled in this study), Oe and Oa horizons overlays a humic, partly eluvial AE horizon and a bleached E horizon, followed by Bh and Bs horizons. Ground vegetation is dominated by *Deschampsia flexuosa* L. Trin. and mosses (Gundersen and Rasmussen 1995). The managed forest stand is the second generation after heathland conversion and dominated by Norway spruce (Gundersen 1998). Trees were 97 years old in 2014. Bulk atmospheric N deposition averaged 9.4 kg N ha^−1^ y^−1^ from 1988 to 1992.

### Experimental Design

At Alptal, we sampled four pairs of circular plots (20 m^2^ each). These plots were used previously to study N transformations, soil collembola and soil trace gas fluxes (Mohn and others 2000; Hagedorn and others 2001b; Xu and others 2009; Krause and others 2013). Each pair consisting of one control plot and one N addition plot in close vicinity to each other (< 30 m) was selected based on comparable microtopography and vegetation cover. The N addition treatment was assigned randomly to one plot within each pair. Thus, each pair is considered as a block and the experiment was analyzed as a split-block design with four replicate blocks. Nitrogen was added as NH_4_NO_3_ to rainwater collected on a polyethylene sheet spread outside the forest (300 m^2^), which was then directed into a water tank and applied automatically by sprinklers (Schleppi and others 2017). Sprinklers were mounted 1.5 m above ground, so that N was added below canopy level but on top of ground vegetation (Krause and others 2012a). The N treatment was applied during precipitation events (that is, approximately 200 times per year) to mimic natural atmospheric N deposition as realistically as possible without changing the water regime of the plots (Krause and others 2013; Schleppi and others 2017). Control plots received the same amount of unaltered rainwater. In winter, automatic irrigation was replaced by the occasional application of concentrated NH_4_NO_3_ solution on top of snow using a backpack sprayer. Nitrogen addition started in April 1995 and varied annually with the local precipitation regime. At the time of sampling, 21.6 ± 4.6 kg N ha^−1^ y^−1^ (mean ± SD) have been added to N addition plots (Krause and others 2012a).

At Klosterhede, the original experimental design involved one N addition area (15 m × 15 m) and two control areas (15 m × 15 m and 15 m × 10 m) located side by side to the N addition area (Gundersen and Rasmussen 1995). The treatment area had received N in the form of NH_4_NO_3_ at a rate of 35 kg N ha^−1^ y^−1^ since February 1992 by hand-spraying of monthly aliquots (except for 2 years 1998–1999 and in a few drought periods). Water added to the N treated area was less than 1% of the throughfall volume, while control areas were subjected to natural precipitation (Gundersen and Rasmussen 1995). For the present study, we divided each area (N addition and combined controls) into four plots (7.5 m × 7.5 m), which are regarded as experimental units in the statistical analysis (detailed below). Then, each N addition plot was paired with the control plot in closest juxtaposition to create four blocks of two plots each (Figure S7.1). Due to the original layout of the experiment, N addition plots were spatially segregated. However, the arrangement of control plots allowed us to test whether spatial gradients were present across the experimental area using multivariate ordination techniques (see Appendix S7 for details). As we did not find such gradients, we analyzed the experiment as a split-block design.

### Soil Sampling

We collected genetic soil horizons from three sampling locations within each plot (that is, a total of 12 samples for each horizon/treatment combination). Sampling locations were selected in the field to cover topographic variation within each plot. As microtopography varies strongly over short distances at Alptal, we collected samples from both mounds (17 locations) and depressions (7 locations) and included microtopography as a random factor in statistical analysis. At each sampling location, organic horizon material was quantitatively removed from within a 25 cm × 25 cm metal frame and horizon depth was measured at each side of the pit. Visible roots were separated by hand and weighed, before the fresh mass of organic material was determined on site. It is important to note that we did not separate live and dead roots, which has implications for our definition of FRB (see ‘Discussion’). Subsequently, four cores of mineral soil were sampled with a steel corer (length 30 cm, diameter 4.5 cm). Depths of mineral horizons were determined on each retrieved core before individual horizons were pooled to create one composite sample per horizon for each sampling location. Fresh mass of each composite sample was determined on site. Soil and root samples were stored in airtight plastic bags, placed in cooled boxes and immediately transported to the laboratory. Sampling was conducted from April 23 to 24, 2014 (Klosterhede), and from June 10 to 12, 2014 (Alptal).

Upon arrival in the laboratory, small roots that were not visible in the field were separated from organic soil samples by hand. Then, root-free organic soil material was ground in a polytron blender (7000 rpm for 3 min; Retsch Grindomax GM2000, Retsch, Haan, Germany). Although grinding does not affect bulk chemical properties, salt-extractable C and N pools and potential C mineralization rates might have been affected. Samples from N addition and control plots, however, were treated in the exact same manner allowing for comparisons between treatments.

Mineral soil samples were sieved through a 2 mm mesh to retain roots, stones and particulate organic matter that remained on the sieve. All roots were sorted by hand under a binocular into fine and coarse roots using a diameter cutoff of 2 mm. Masses of roots, particulate organic matter and stones were determined before and after oven-drying (70°C, 24 h). Gravimetric water content and soil dry mass were determined by drying soil subsamples at 105°C to constant mass. Another set of subsamples was air-dried to constant mass for the analyses of soil texture, exchangeable cations, carbonate and concentrations of C and N. The remaining field-moist soil was stored at 4°C up to two weeks for analysis of K_2_SO_4_-extractable C and N pools, soil pH and potential C mineralization rates.

### Physicochemical Soil Properties

The texture of mineral horizons was determined using the pipette method according to standard ÖNORM procedures (www.austrian-standards.at/en). For selected horizons, particle size distribution was additionally quantified with a sedigraph (SediGraph III, micromeritics Germany GmbH, Aachen, Germany) after organic matter had been oxidized with H_2_O_2_. Data from the pipette method are shown for Klosterhede and from the sedigraph method for Alptal. Soil pH was electrochemically measured in 1:10 w/v slurries of soil and Milli-Q water (WTW 196, WTW, Bayern, Germany). Exchangeable cations were determined in unbuffered BaCl_2_ extracts (ÖNORM L 1086-1) via atomic absorption spectroscopy (for Ca^2+^, Mg^2+^, K^+^, Na^+^, Fe^3+^, Mn^2+^; PinAAcle 900T, Perkin Elmer, MA, USA) or ICP-MS (for Al^3+^; 7700×, Agilent Technologies Österreich, Vienna, Austria). Effective cation exchange capacity (CEC_eff_) was calculated as the sum of the above cations, while base saturation (BS_eff_) represents the fractional contribution of base cations (Ca^2+^, Mg^2+^, K^+^ and Na^+^) to CEC_eff_. Ammonium-N and NO_3_^−^-N were determined photometrically (Perkin Elmer 2300 EnSpire, USA) in 0.5 M K_2_SO_4_ extracts (1:10 w/v for mineral horizons, 1:20 w/v for organic horizons) using published methods (Hood-Nowotny and others 2010). Concentrations of non-purgeable organic carbon (NPOC) and total dissolved nitrogen (TDN) were determined in K_2_SO_4_ extracts using a TOC/TN analyzer (Shimadzu TOC-L/TNM-L, Shimadzu, Korneuburg, Austria). Non-purgeable organic C was taken as a measure of extractable organic carbon (EOC). Extractable organic nitrogen (EON) was calculated by subtracting the sum of extractable NH_4_^+^-N and NO_3_^−^-N from TDN.

### Carbon and Nitrogen Pools of Soil and Fine Roots

Subsamples of air-dried soil were ground (1500 rpm for 2.5 min, MM2000, Retsch, Haan, Germany) and analyzed for total C and N concentrations by dry combustion at 1050°C (Carlo Erba NA 1500, Milan, Italy) according to standard ÖNORM procedures (www.austrian-standards.at/en). Inorganic C was measured as CO_2_ after treatment with 10% HCl by the Scheibler method and subtracted from total C to obtain SOC concentrations. Soil organic C pools (SOCP; kg m^−2^) were calculated for each horizon *i* as

1 $$ {\text{SOCP}}_{i} = \rho_{{{\text{B}},i}} \times d_{i} \times {\text{SOC}}_{i} \times \left( {1 - \left( {\theta_{i} /100} \right)} \right), $$

where *ρ*_B*,i*_ is fine earth bulk density (kg m^−3^), *d*_*i*_ is horizon thickness (m), SOC_*i*_ is SOC concentration (kg kg^−1^), and *θ*_*i*_ is volume fraction of roots and stones. Soil total N pools (STNP) were calculated by substituting total N concentration (STN_*i*_) for SOC_*i*_ in equation (1). Our method for calculating SOC and STN pools essentially equals method 4 of Poeplau and others (2017) as we corrected estimates of soil volume for stone volume. In addition, we also corrected soil volume for the volume of roots (see Appendix S4 for details on calculations of *ρ*_B,*i*_ and *θ*_*i*_).

We recalculated mineral horizon SOC pools in 10-cm increments as

2 $$ {\text{SOCP}}_{\text{increment}} = \mathop \sum \limits_{i = 1}^{k} \left( {{\text{SOCP}}_{i} \times F_{i} } \right), $$

where *k* is the number of genetic mineral soil horizons within the respective depth increment, SOCP_*i*_ is the C pool of genetic mineral soil horizon *i* (kg m^−2^), and *F*_*i*_ is the fraction of genetic mineral soil horizon *i* within the respective depth increment. Mineral horizon STN pools were recalculated accordingly. To account for the influence of horizon thickness on pools, we also calculated pools sizes of modeled, 1-cm-thick layers for each horizon (kg m^−2^ cm^−1^; Müller and Kögel-Knabner 2009):

3 $$ {\text{SOCP}}_{{{\text{modeled}},i}} = \frac{{{\text{SOCP}}_{i} }}{{\left( {d_{i} \times 100} \right)}}. $$

Modeled STN pools were calculated accordingly.

Subsamples of dried fine roots were milled (MM2000, Retsch, Haan, Germany) before C and N concentrations were determined by dry combustion at 1250°C (LECO TruSpec CN, LECO, Mönchengladbach, Germany). Fine root C pools (FRCP) for each horizon *i* (g m^−2^) were calculated as

4 $$ {\text{FRCP}}_{i} = \frac{{M_{i} }}{{A_{i} }} \times {\text{FRC}}_{i} \times 10^{3} , $$

where *M* is fine root dry mass (kg), *A* is sampling area (m^2^), and FRC is fine root C concentration (kg kg^−1^). Fine root N pools (FRNP) were calculated accordingly.

### Potential C Mineralization Rates and Ecosystem-Level Response to N Addition

Potential rates of C mineralization were measured in short-term laboratory incubations to evaluate if long-term N addition affected C availability in the soil (see Appendix S6 for details). To assess the ecosystem-level response to N addition, we further compiled data on tree growth, needle biomass, needle chemistry and litter N concentrations (see Figure 3 legend for references). Nitrogen effects on ecosystem C cycling were assessed by summarizing related parameters such as foliar litterfall or soil CO_2_ efflux from published and unpublished sources (see Tables S6.1, S6.2 for complete list of parameters including references).

### Data Analysis

We used linear (mixed) models in R (v3.2.5) to test for the fixed effects of N treatment, soil horizon and their interaction on soil and fine root pools as well as on physicochemical soil properties (R Core Team 2016). Data were tested for normality and homogeneity of variances using Shapiro–Wilk and Levene’s tests, respectively. In case of non-normality or heteroscedasticity, data were log- or box-cox-transformed using the ‘powerTransform’ function from the ‘car’ package (Fox and Weisberg 2011). When sufficient replicates were available to allow for the inclusion of random effects (that is, for pools and most physicochemical soil properties), we built linear mixed models using the packages ‘lme4’ (Bates and others 2015) and ‘afex’ (Singmann and others 2015). Block and replicate within block and treatment were included as random effects in the models to account for spatial autocorrelation. Microtopographic position (mounds/depressions) was included as random effect for Alptal. Post hoc differences between soil horizons were assessed by Tukey’s HSD tests, and Dunnett’s tests were used to test for differences between treatments within horizons using the ‘lsmeans’ package (Lenth 2016).

Low replication precluded the inclusion of random effects for some variables (sand/silt/clay, CEC_eff_, BS_eff_). In these cases, we built simple linear models (package ‘stats’) and tested for effects of N treatment, soil horizon and their interaction with Type III ANOVA as implemented in the ‘car’ package. Differences between soil horizons were assessed using pairwise contrasts with Tukey’s HSD tests (package ‘lsmeans’). Assumptions were checked visually for all models by plots of residuals vs. fitted values and qq-plots. For some variables, model residuals severely violated normality or heteroscedasticity even after data transformation (concentrations of SOC, STN, NH_4_^+^-N, NO_3_^−^-N, EON at Klosterhede). Consequently, we split the data set and reran the analyses separately for organic and mineral horizons (package ‘lmerTest’; Kuznetsova and others 2017). Simple linear regression was used to test for linear relationships between calendar year and N-induced changes in basal area increment (BAI), average needle biomass, needle Mg/N and litter N (Figure 3). In addition, median responses of N concentrations of fine roots and soil were added to Figure 3. We did not statistically evaluate the effects of N addition on ecosystem C cycling due to limited data availability.

## Results

### Physicochemical Soil Properties

**Figure 1:**
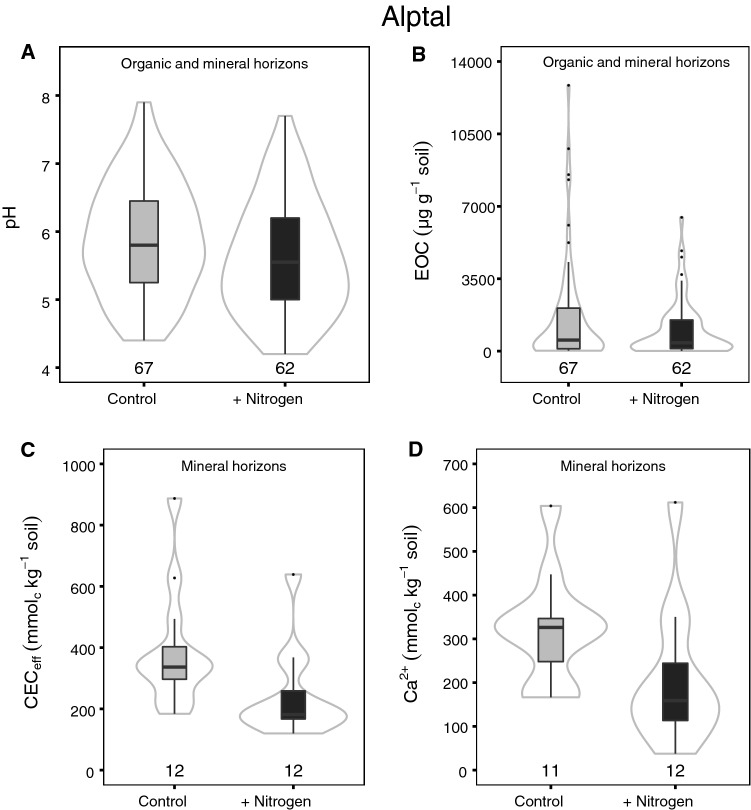
Soil pH (**A**), extractable organic C (**B**), cation exchange capacity (**C**) and exchangeable calcium (**D**) across all horizons (**A**, **B**) and in mineral horizons (**C**, **D**) at Alptal. *Box–whisker plots* are shown for each treatment. The *black line* is the median, *lower* and *upper* boundaries correspond to the first and third quartiles, respectively, and whiskers span 1.5 times the interquartile range. *Gray lines* around the boxes depict Gaussian kernel estimates of probability densities. Sample numbers are depicted above the *x*-axes.

Two decades of N addition consistently reduced soil pH at Alptal by 0.28 across all horizons (*P* = 0.032, Figure 1A, Table S1.1). Extractable organic carbon (EOC) concentrations were marginally lower in N addition plots by 38% on average across all horizons (*P* = 0.089, Figure 1B, Table S1.1). Similarly, N addition decreased CEC_eff_ in mineral horizons by 38% (*P* = 0.010, Figure 1C), mainly due to loss of exchangeable calcium (*P* = 0.008, Figure 1D, Table S1.2). Nitrogen addition also increased extractable NH_4_^+^ concentrations in the Oe horizon (*P* = 0.043), as well as extractable NO_3_^−^ concentrations in Oi and Oa horizons (*P* = 0.021 and *P* = 0.050, respectively), whereas extractable inorganic N pools in mineral soil horizons were not affected (Table S1.1).

**Figure 2:**
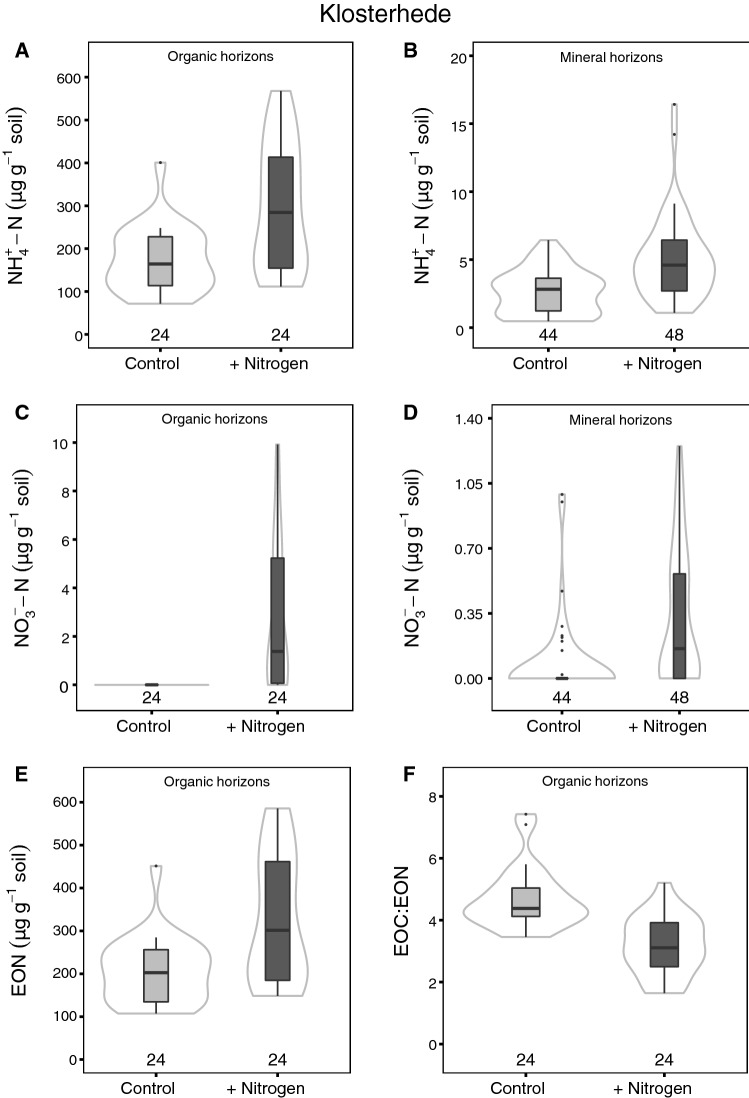
Salt-extractable pools of NH_4_^+^-N (**A**, **B**), NO_3_^−^-N (**C**, **D**), organic N (**E**) and salt-extractable EOC/EON (**F**) in organic horizons (**A**, **C**, **E**, **F**) and mineral horizons (**B**, **D**) at Klosterhede. *Box–whisker plots* are shown for each treatment. The *black line* is the median, *lower* and *upper* boundaries correspond to the first and third quartiles, respectively, and whiskers span 1.5 times the interquartile range. *Gray lines* around the boxes depict Gaussian kernel estimates of probability densities. Sample numbers are depicted above the *x*-axes.

Nitrogen addition had even more pronounced effects on extractable inorganic N pools at Klosterhede. Ammonium was consistently higher across the whole soil profile in N addition plots (*P* < 0.001 in both organic and mineral horizons; Figure 2A, B, Table S1.3). In comparison, NO_3_^−^ was often not detectable in control plots, but low concentrations were measured in N addition plots (*P* < 0.001 and *P* = 0.002 in organic and mineral horizons, respectively; Figure 2C, D, Table S1.3). Likewise, EON was significantly higher in organic horizons (*P* < 0.001, Figure 2E) as well as in AE and E horizons of N addition plots (*P* = 0.001 and *P* = 0.009, respectively; Table S1.3). There was a significant interaction of N addition treatment and horizon on EOC/EON (*P* = 0.011), with smaller ratios under high N inputs in the organic layer (Figure 2F) and AE horizon and similar ratios in mineral subsoil (Table S1.3). Nitrogen addition did not affect soil pH or CEC_eff_ at Klosterhede (Tables S1.3, S1.4).

### Tree Growth and N Accumulation Patterns

**Figure 3:**
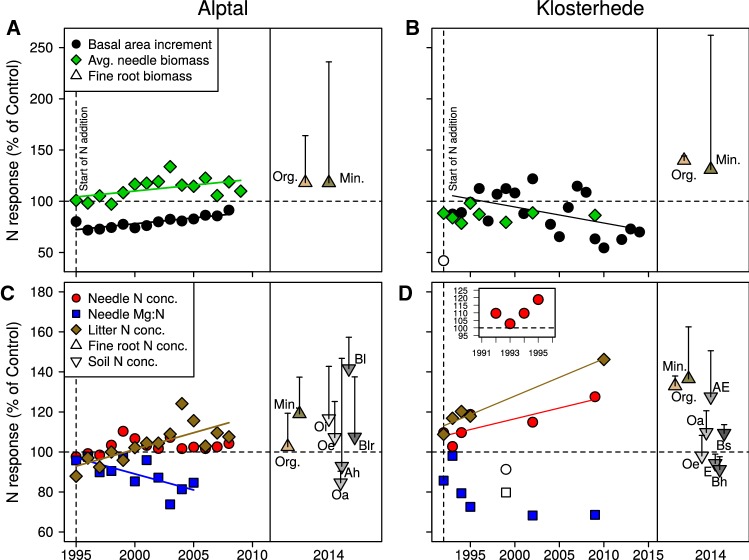
Relative response of tree growth and N accumulation in various ecosystem compartments to two decades of N addition at Alptal (**A**, **C**) and Klosterhede (**B**, **D**). **A**, **B** show tree growth (basal area increment), average needle biomass and fine root biomass. **C**, **D** show N concentrations in needles, litter, fine roots and soil, and needle Mg/N. The insert in (**D**) shows needle N concentrations at Klosterhede from 1992 to 1995. *Symbols* for fine root biomass, fine root N and soil N concentrations represent median responses, a more robust measure of location than means. *Error bars* are the adjusted median absolute deviations (MAD), a robust measure of dispersion analogous to standard deviation (Huber and Ronchetti 2009; Leys and others 2013). Assuming normal distribution of the data, the adjusted MAD encompasses 50% of the observations (Rousseeuw and Croux 1993). Fine root biomass and N concentrations were summarized for organic (‘Org.’) and mineral (‘Min.’) horizons. For soil horizon designation see Table 1. Data on basal area increment, needle biomass, needle N concentrations and needle Mg/N and, in part, litter N concentrations have been obtained from Ginzburg (2014), Gundersen (1998), Krause and others (2012a, 2013), P. Gundersen (unpublished results) and P. Schleppi (unpublished results). For data on fine root biomass and fine root N concentrations see Appendix S3. For data on soil N concentrations see Appendix S4. Note that fine root biomass, fine root N concentrations and soil N concentrations were measured on two occasions in 2014 and are separated graphically in plots to enhance readability. The linear regression equations for basal area increment, needle biomass, needle Mg/N and litter N concentrations are shown in Table S2.1. Open symbols depict points excluded from regression analyses.

Long-term effects of N addition on tree growth, needle biomass, needle chemistry and litter N concentrations were assessed using time series data (Figure 3). Tree growth was differently affected by N additions at the two sites. At Alptal, basal area increment (BAI) increased by 1.2% per year in response to N between 1995 and 2008 (Figure 3A; see Table S2.1 for regression statistics). Similarly, N addition increased average needle biomass by 1.2% per year relative to controls between 1995 and 2009 (Figure 3A, Table S2.1). At Klosterhede, BAI declined by 1.6% per year in response to high N inputs between 1993 and 2013 (Figure 3B, Table S2.1), while average needle biomass was not affected (Figure 3B). Along with the reduction in BAI, tree mortality was higher in N addition plots at Klosterhede (P. Gundersen, pers. comm.).

At Alptal, N addition induced a relative accumulation of N in litter (+ 1.7% per year over controls between 1995 and 2008, Figure 3C); response of needle N concentrations, however, was only transient (Figure 3C, Krause and others 2012a). At Klosterhede, needle N concentrations in N addition plots increased by 1.1% per year relative to controls between 1992 and 2009 (Figure 3D). Similarly, litter N concentrations increased by 1.9% per under high N inputs between 1992 and 2010 (Figure 3D). Median N concentrations of fine roots were higher with N in organic and mineral horizons (+ 33 and + 37%, respectively) and so were median STN concentrations of Oa, AE and Bs horizons (Figure 3D).

### Fine Roots

The horizon-specific analysis revealed that N addition had little effect on fine root traits at Alptal. Fine root biomass did not respond to N addition except for the Blr horizon (horizon/treatment interaction *P* = 0.022), where we observed a significant, but small increase in FRB (*P* < 0.001, Figure S3.1). Also, N addition increased FRN concentrations only in the Ah horizon (+ 26%, *P* = 0.016) where fine root C/N was reduced accordingly (− 21%, *P* = 0.011, Table S3.1). Overall, FRC and FRN pools were not affected by N at Alptal (Figure S3.2).

**Figure 4:**
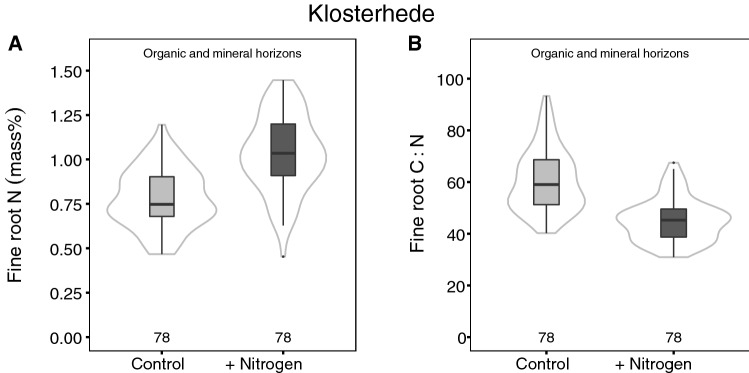
Fine root N concentrations (**A**) and fine root C/N (**B**) across all horizons at Klosterhede. *Box–whisker plots* are shown for each treatment. The *black line* is the median, *lower* and *upper* boundaries correspond to the first and third quartiles, respectively, and whiskers span 1.5 times the interquartile range. *Gray lines* around the boxes depict Gaussian kernel estimates of probability densities. Sample numbers are depicted above the *x*-axes.

**Figure 5:**
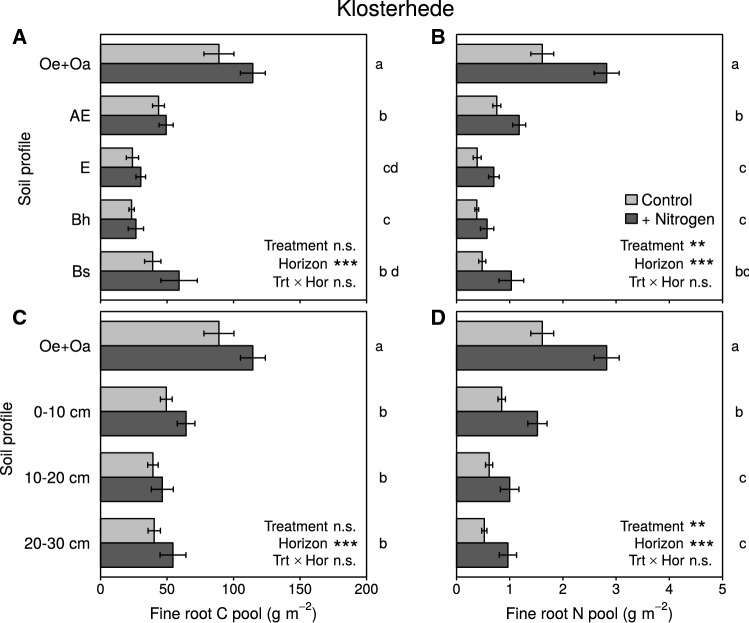
Fine root C pools (**A**, **C**) and fine root N pools (**B**, **D**) in genetic horizons (*upper panels*) and depth increments (*lower panels*) at Klosterhede. Means (± SE) were derived from 12 samples except for the following combinations of horizon/increment and treatment: Bh Control, Bs Control, 10–20 cm Control and 20–30 cm Control (10 samples). Lower-case letters right to each plate originate from pairwise comparison of horizon/increment means. Means with no letter in common are significantly different (Tukey’s HSD, *α* = 0.05). The N addition main effects for fine root N pools were significant at *P* = 0.010 and *P* = 0.006 for genetic horizons (**B**) and depth increments (**D**), respectively.

In contrast, fine roots were strongly affected by N addition at Klosterhede. Fine root biomass and FRN concentrations increased in response to N across all horizons (FRB: + 12 to + 86%, *P* = 0.016; FRN concentrations: + 19 to + 46%, *P* < 0.001; fine root C/N: − 16 to − 32%, *P* < 0.001; Figure 4A, B, Figure S3.3, Table S3.2). As a result, N addition significantly increased whole-profile FRC pools (*P* = 0.038, Figure 5A, C, Table S5.1). Fine root N pools were consistently higher in N addition plots across all soil horizons (*P* < 0.001, Figure 5B, D, Table S5.1), due to of both higher FRB and higher FRN concentrations.

### Soil Organic C and Soil Total N Pools

**Figure 6:**
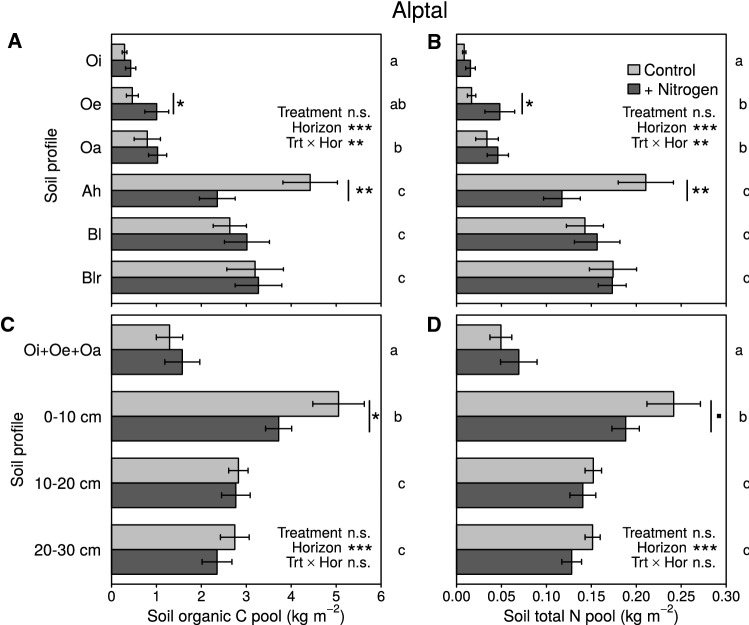
Soil organic C pools (**A**, **C**) and soil total N pools (**B**, **D**) in genetic horizons (*upper panels*) and depth increments (*lower panels*) at Alptal. Means (± SE) were derived from 12 samples except for the following combinations of horizon and treatment: Oi + N (11 samples), Oe Control (7), Oe + N (8), Oa Control (11), Oa + N (6), Ah Control and Ah + N (13). Lower-case letters right to each plate originate from pairwise comparison of horizon/increment means. Means with no letter in common are significantly different (Tukey’s HSD, *α* = 0.05). *Symbols* next to *bars* indicate significant post hoc differences between treatments within a given horizon/increment (^■^*P* < 0.1; *P < 0.05; **P < 0.01; ***P < 0.001). Note that post hoc differences were found for pools of 0–10 cm increments (**C**, **D**) despite nonsignificant interactions.

We found significant interactions between horizon and N addition for SOC and STN pools at both sites (Alptal: *P* = 0.007 for both SOC and STN pools; Klosterhede: SOCP *P* = 0.028, STNP *P* = 0.099). At Alptal, SOC pools in the Oe horizon were 120% larger in N addition plots (1.01 vs. 0.46 kg m^−2^ in controls, *P* = 0.039, Figure 6A). In contrast, Ah horizon SOC pools were 47% smaller in N addition plots compared to controls (2.35 vs. 4.42 kg m^−2^, *P* = 0.002, Figure 6A). Similarly, STN pools were 150% larger in the Oe horizon (0.05 vs. 0.02 kg m^−2^, *P* = 0.021, Figure 6B) and 44% smaller in Ah horizons in the N addition plots (0.12 vs. 0.21 kg m^−2^, *P* = 0.0002, Figure 6B). The effect of N addition on SOC and STN pools was, however, smaller when fixed-depth increments were compared at both sites (increment/treatment interactions for Alptal: SOCP *P* = 0.102, STNP *P* = 0.129; for Klosterhede: SOCP *P* = 0.159, STNP *P* = 0.456). At Alptal, SOC and STN pools of combined O horizons still tended to be larger in N addition plots (+ 22 and + 40%, respectively), but the effects of N addition treatment were no longer significant (*P* = 0.298 and *P* = 0.184, respectively; Figure 6C, D). In the 0–10 cm depth increment, SOC and STN pools were 26 and 22% smaller in N addition than in control plots, respectively (*P* = 0.031 and *P* = 0.055, respectively; Figure 6C, D). These N-induced shifts in SOC and STN pools at Alptal were also apparent when sampling sites located in depressions were excluded from the analysis (data not shown).

**Figure 7:**
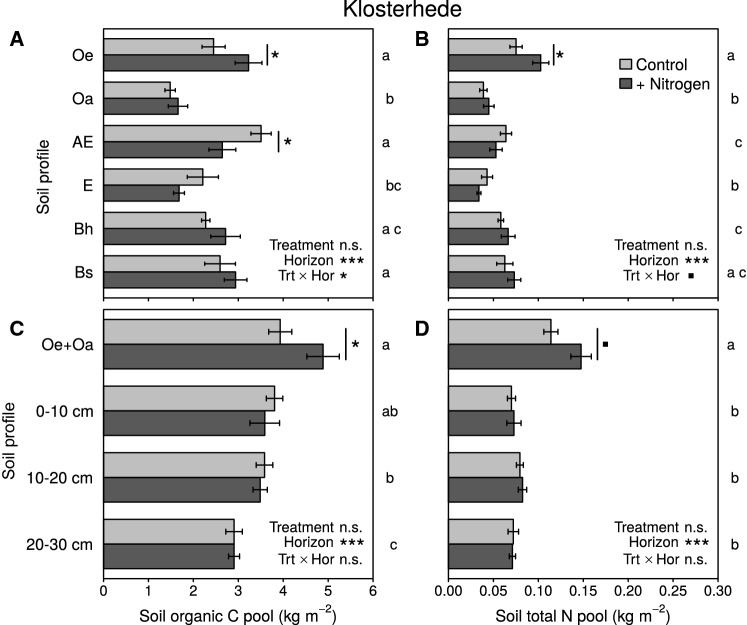
Soil organic C pools (**A**, **C**) and soil total N pools (**B**, **D**) in genetic horizons (*upper panels*) and depth increments (*lower panels*) at Klosterhede. Means (± SE) were derived from 12 samples except for the following combinations of horizon/increment and treatment: Bh Control, Bs Control, 10–20 cm Control and 20–30 cm Control (10 samples each). Lower-case letters right to each plate originate from pairwise comparison of horizon/increment means. Means with no letter in common are significantly different (Tukey’s HSD, *α* = 0.05). *Symbols* next to *bars* indicate significant post hoc differences between treatments within a given horizon/increment (^■^*P* < 0.1; *P < 0.05; **P < 0.01; ***P < 0.001). Note that post hoc differences were found for pools of combined organic horizons (**C**, **D**) despite nonsignificant interactions.

At Klosterhede, the SOC pool in the Oe horizon was 32% larger in N addition plots (3.23 vs. 2.45 kg m^−2^ in controls, *P* = 0.043), whereas N inputs reduced the AE horizon SOC pool by 25% (2.64 vs. 3.51 kg m^−2^ in controls, *P* = 0.024, Figure 7A). Moreover, the STN pool in the Oe horizon was 37% larger in N addition plots (0.10 vs. 0.08 kg m^−2^ in controls, *P* = 0.025, Figure 7B). Pools of SOC and STN summed across organic horizons were higher in N addition plots compared to controls (SOCP: 4.89 vs. 3.93 kg m^−2^, *P* = 0.037; STNP: 0.15 vs. 0.11 kg m^−2^, *P* = 0.063; Figure 7C, D). Soil organic C and STN pools in subsoil horizons below 10 cm depth were not affected by the N addition treatment neither at Alptal nor at Klosterhede (Figures 6, 7).

### Horizon Thickness, Bulk Density and Elemental Concentrations of Genetic Soil Horizons

Higher SOC pools in the Oe horizon of N addition plots at Alptal were due to greater soil masses rather than higher SOC concentrations. Oe horizons in N addition plots were insignificantly thicker (+ 55%, *P* = 0.179) and had a higher bulk density (+ 73%, *P* = 0.033) compared to control plots, resulting in greater masses of Oe horizons (*P* = 0.014), while neither SOC nor STN concentrations were significantly affected by N (Table S4.1). However, N addition significantly reduced SOC/STN of Oe horizon material from 27 to 23 (*P* = 0.033, Table S4.1). Conversely, thickness and bulk density of the Ah horizon tended to be lower in N addition plots compared to controls (− 19 and − 17%, respectively; *P* = 0.119 and *P* = 0.163). Soil organic C concentrations of the Ah horizon were lower in N addition plots (− 24%, *P* = 0.039), and STN concentrations tended to decrease in N addition plots as well (− 20%, *P* = 0.095, Table S4.1).

At Klosterhede, both horizon thickness and bulk density of the Oe horizon tended to be higher in N addition plots (+ 8 and + 20%, respectively; Table S4.2). In contrast, N addition significantly reduced thickness and bulk density in the AE horizon by 24% (*P* = 0.016) and 12% (*P* = 0.011), respectively (Table S4.2). Nitrogen addition further tended to increase SOC concentrations of the Oa horizon (+ 5%, *P* = 0.062) and increased STN concentrations in the Oa horizon (+ 9%, *P* = 0.034, Table S4.2). Similarly, STN concentrations of the AE horizon tended to be higher in N addition plots (+ 25%, *P* = 0.098), resulting in a marginally significant decline in SOC/STN from 57 to 52 (*P* = 0.094, Table S4.2).

We further estimated the relative importance of horizon thickness and bulk density for pool sizes by calculating horizon-specific pools for modeled, 1-cm-thick layers (Tables 2, 3). At Alptal, N-induced increases in Oe horizon SOC pools as well as decreases in Ah horizon SOC pools prevailed after accounting for differences in horizon thickness. Thus, changes in pool sizes were mainly controlled by corresponding changes bulk density (Oe horizon) or SOC concentrations (Ah horizon, Table S4.3). At Klosterhede pool sizes of modeled, 1-cm-thick layers did not differ between N treatments (Table 3).

**Table 2:** Soil Organic C and Soil Total N Pools of Modeled, 1-cm-Thick Layers at Alptal

	SOC pool (kg m^−2^ cm^−1^)	STN pool (kg m^−2^ cm^−1^)
Oi	*a*	*a*
Control	0.27 ± 0.04	***0.008 ± 0.001***
+ Nitrogen	0.39 ± 0.09	***0.013 ± 0.004***
Oe	*ab*	*ab*
Control	***0.27 ± 0.06***	**0.010 ± 0.002**
+ Nitrogen	***0.40 ± 0.07***	**0.018 ± 0.004**
Oa	*ab*	*ab*
Control	**0.22 ± 0.04**	**0.009 ± 0.002**
+ Nitrogen	**0.40 ± 0.06**	**0.017 ± 0.004**
Ah	*b*	*c*
Control	**0.55 ± 0.07**	**0.026 ± 0.003**
+ Nitrogen	**0.36 ± 0.04**	**0.018 ± 0.002**
Bl	*a*	*bc*
Control	0.28 ± 0.02	0.015 ± 0.001
+ Nitrogen	0.33 ± 0.04	0.017 ± 0.002
Blr^a^	a	b
Control	0.28 ± 0.03	0.015 ± 0.001
+ Nitrogen	0.23 ± 0.03	0.013 ± 0.001
Sign. effects	Horizon, + N × horizon	+ N^b^, horizon, + N × horizon

Bold and bold italic values indicate significant (*P* < 0.05) and marginally significant (*P* < 0.1) differences between treatments, respectively. Means (± SE) were derived from 12 samples except for the following horizon/treatment combinations: Oi + N (11 samples), Oe Control (7), Oe + N (8), Oa Control (11), Oa + N (6), Ah Control and Ah + N (13). Lower-case letters within columns indicate post hoc differences between horizons (*P* < 0.05).

^a^Blr horizons were sampled to an average depth of 30.0 ± 2.1 and 26.3 ± 1.0 cm from top of mineral soil in control and N addition plots, respectively.

^b^Marginally significant main effect (*P* = 0.088).

**Table 3:** Soil Organic C and Soil Total N Pools of Modeled, 1-cm-Thick Layers at Klosterhede

	SOC pool (kg m^−2^ cm^−1^)	STN pool (kg m^−2^ cm^−1^)
Oe	*a*	*a*
Control	0.49 ± 0.04	0.015 ± 0.001
+ Nitrogen	0.59 ± 0.04	0.019 ± 0.001
Oa	a	ab
Control	0.60 ± 0.09	0.016 ± 0.003
+ Nitrogen	0.51 ± 0.06	0.014 ± 0.002
AE	a	c
Control	0.42 ± 0.02	0.007 ± 0.000
+ Nitrogen	0.41 ± 0.04	0.008 ± 0.001
E	b	d
Control	0.29 ± 0.03	0.006 ± 0.000
+ Nitrogen	0.24 ± 0.01	0.005 ± 0.000
Bh	a	b
Control	0.45 ± 0.02	0.012 ± 0.001
+ Nitrogen	0.44 ± 0.02	0.011 ± 0.001
Bs^a^	b	c
Control	0.27 ± 0.02	0.007 ± 0.001
+ Nitrogen	0.28 ± 0.01	0.007 ± 0.000
Sign. effects	Horizon	Horizon

Means (± SE) were derived from 12 samples except for the following horizon/treatment combinations: Bh Control, Bs Control, 10–20 cm Control and 20–30 cm Control (10 samples). Lower-case letters within columns indicate post hoc differences between horizons (*P* < 0.05).

^a^Bs horizons were sampled to an average depth of 31.6 ± 1.3 and 29.7 ± 0.8 cm from top of mineral soil in control and N addition plots, respectively.

### Total Belowground C and N Pools

Nitrogen addition had no effects on soil, fine root or total belowground (soil plus fine root) pools of C or N at Alptal (Table S5.1). In contrast, N addition marginally increased the STN pool down to 30 cm at Klosterhede by 15% (*P* = 0.055). Consequently, total belowground C pools did not change with N at Klosterhede, while total belowground N pools were increased by 13% on average (*P* = 0.044, Table S5.1).

### Potential C Mineralization Rates and Ecosystem-Level C Cycling

Carbon mineralization rates did not differ between N addition and control plots in laboratory incubations, except for the Oe horizon where potential C mineralization per g SOC was increased by 144% (*P* = 0.001, Figure S6.1). The compilation of long-term monitoring data revealed site-specific patterns of ecosystem-level C cycling in response to N (see Figure S6.2, Tables S6.1, S6.2). At Alptal, aboveground C inputs to soil generally increased with N addition as trees grew better and produced more needle litter (+ 22%) over 13 years of N treatment. Conversely, C outputs via in situ soil CO_2_ efflux tended to decrease (− 18%), although high between-plots variability precluded the detection of a significant N effect (Krause and others 2013). At Klosterhede, aboveground C inputs via litterfall showed little response to increased N inputs. There was, however, a tendency toward reduced growth of trees and mosses with N addition (− 12 and − 78%, respectively; Table S6.2). Belowground C inputs from roots and ectomycorrhiza tended to be lower in N addition plots while C outputs via litter decomposition or soil CO_2_ efflux were not affected whether measured in situ or the laboratory (see Table S6.2 for references).

## Discussion

Experimental N addition was observed to increase SOC pools in temperate and boreal forests (Högberg and others 2006; Pregitzer and others 2008; Lovett and others 2013; Frey and others 2014; Maaroufi and others 2015). In contrast to these findings and our first hypothesis, we found that whole-profile SOC pools were not affected by two decades of regular, low-dose N addition at the investigated sites. Although higher N inputs led to a SOC accumulation in Oe horizons, this was offset by a concomitant reduction of SOC pools in A horizons. Consequently, N addition did not affect total SOC pools down to 30 cm depth, but resulted in a vertical redistribution of SOC within the soil profile, with gains in organic horizons and losses in mineral topsoil horizons. Further, FRB was either unresponsive to or increased with additional N at Alptal and Klosterhede, respectively, which contradicts our second hypothesis based on negative N responses of FRB observed in gradient studies and N manipulation experiments (for example, Li and others 2015).

### Experimental N Addition in Relation to Background N Deposition

One of the reasons for the relatively small responses of belowground C pools to long-term N addition might be the modest relative increase in N inputs. At both sites, the amount of experimentally added N was relatively low compared to annual background N deposition (N addition/throughfall N deposition: 1.3 at Alptal, 1.5 at Klosterhede). Two reasons can be given for these low ratios. First, rates of experimental N addition were chosen to be well below doses applied for forest fertilization (for example, Hyvönen and others 2008) to realistically mimic elevated inputs from the atmosphere (Galloway and others 2008; Simpson and others 2014). Second, measured throughfall N deposition rates were already relatively high at the both sites, the late 1980s and early 1990s (17 and 23 kg N ha^−1^ y^−1^ at Alptal and Klosterhede, respectively; Table 1). Since then, N deposition has slightly declined throughout Europe, but atmospheric input rates are still higher than in large parts of North America (Vet and others 2014; Jia and others 2016). Notwithstanding the similarities in N addition/throughfall, Klosterhede received about 60% more additional N than Alptal during the study period. This might have contributed to the divergent responses observed at Klosterhede (for example, reduced tree vigor or belowground N accumulation) than at Alptal.

### Nitrogen Addition Induced Either Soil Acidification or Belowground N Accumulation, Depending on Site

Experimentally added N affected soil pH, exchangeable cations and belowground N pools, all of which can alter SOC storage via effects on tree productivity (Oren and others 2001; Högberg and others 2006; Goll and others 2012) and decomposition of SOM (Sollins and others 1996; Hobbie and others 2007). Our results show that these changes were highly site-specific, emphasizing the importance of inherent site characteristics (Lu and others 2015) in addition to the aforementioned differences in N application and deposition rates. Although experimental N addition reduced both soil pH and exchangeable cations (mainly calcium) in the pedogenetically ‘younger’ soil at Alptal (Figure 1; Xu and others 2009), these properties were not affected by N in the pedogenetically ‘older’ soil at Klosterhede. This suggests that the Gleysol at Alptal was able to physicochemically buffer the effects of N addition more effectively than the Podzol at Klosterhede where the soil was already strongly acidic in the control plots. Even though Klosterhede is situated close to the sea and receive cations via sea salt spray, CEC_eff_ in the control plots was two orders of magnitude lower than at Alptal, which might have limited further cation losses in response to N addition.

Declines in soil pH and loss of base cations are well-known consequences of N addition (Tamm 1991; McNulty and Aber 1993; Högberg and others 2006; Lucas and others 2011; Tian and Niu 2015). Both effects were observed at Alptal and have been shown to impede nutrient uptake by trees (Schröder and others 1988; Schulze 1989), and cause elemental imbalances in foliage (Schaberg and others 1997; Gundersen 1998; Jonard and others 2015), which ultimately might limit plant productivity and C inputs to soil. At Alptal, however, tree growth positively responded to N until 2008 (Figure 3). Thus, N-induced declines in soil pH and exchangeable cations at this site with carbonate-containing parent material (Hagedorn and others 2001b) were apparently not severe enough to outweigh the fertilizing effect of N on trees.

At Klosterhede, increases in extractable N and fine root N after two decades of experimental N addition point to a different response trajectory (Figures 2, 4). Increases in extractable inorganic N pools in response to N addition have been commonly observed (Lu and others 2011a) and are in line with results from Klosterhede after four treatment years (Gundersen 1998). In addition, substantial amounts of added N were taken up by trees at this site (Gundersen 1998), which increased litter and fine root N concentrations in turn (Figures 3, 4). Thus, the decomposition of plant inputs high in N might have contributed to the observed increases in extractable N pools in organic horizons and mineral topsoil.

In addition, the accumulation of N in labile soil and root pools at Klosterhede may have contributed to increased SOC storage in the Oe horizon by decelerating SOM decomposition through several mechanisms. First, inorganic N can be abiotically incorporated into SOM, which can reduce SOM decomposability (Nömmik and Vahtras 1982; Thorn and Mikita 1992; Berg and Matzner 1997; Compton and Boone 2002). Second, N addition can slow down the decomposition of lignin-rich litter such as spruce needles by increasing litter N concentrations (Figure 3; Knorr and others 2005; Hobbie and others 2012; van Diepen and others 2015). The exact mechanisms behind this slow down are still unclear (van Diepen and others 2015), but might include N-induced changes in efficiency and growth rates of decomposers (Agren and others 2001), in particular of saprotrophic fungi (van Diepen and others 2016), and/or the suppression of lignolytic enzymes (Berg and Matzner 1997). Third, additional N may indirectly slow SOM decomposition by decreasing the mining of soil microorganisms for N (Craine and others 2007; Talbot and others 2008; Sinsabaugh 2010). While litter N concentrations increased as a consequence of N addition, the relative importance of abiotic N incorporation and reduced N mining for C accumulation in the Oe horizon at Klosterhede remains elusive.

### Tree Growth, N Accumulation and the N Saturation Concept

About three decades ago, Aber and others (1989) first proposed that several plant and soil parameters change when temperate forest stands sequentially progress from N limitation to N saturation. While observed plant responses generally fit to this concept of N saturation (Aber and others 1998; Niu and others 2016), its wider applicability beyond plant traits has been questioned (Lovett and Goodale 2011; Niu and others 2016). As a consequence, it has been proposed that a focus on the multiple fates of N (for example, sequestration, denitrification, leaching) might be better suited to address the manifold responses of ecosystems to additional N (Lovett and Goodale 2011). Importantly, this approach distinguishes capacity N saturation, where ecosystem N sinks are zero or negative, from kinetic N saturation, where ecosystem sinks retain N but at lower rates compared to N addition (Lovett and Goodale 2011).

By this definition, both Alptal and Klosterhede quickly approached kinetic saturation as N leaching increased already within the first year of N additions (Gundersen 1998; Schleppi and others 2017). At Klosterhede, NO_3_^−^ leaching was enhanced for 3 years after initiation of N additions although the site had initially been characterized as ‘N-limited’ (Gundersen and others 1998). At Alptal, for which longer measurements are available, leaching of mainly DON and NO_3_^−^ led to a continuous loss of about one-third of the added N during 14 years of treatment (Schleppi and others 2017), indicating that N supply exceeded the retention capabilities of the plant–soil system. Despite high leaching losses, additional N also accumulated in vegetation and soil at both sites (Gundersen 1998; Krause and others 2012a, b), which progressively saturated their N retention capacity. However, the plant–soil system responded differently to additional N at each site (Figure 3).

At Alptal, the sustained, gradual increase in tree growth and needle biomass in the absence of concomitant increases in needle N concentrations suggests that trees accumulated N in parallel with C (Figure 3A, C; Krause and others 2012a). This response corresponds to the C accumulation component of the vegetation sink (Lovett and Goodale 2011) and was linked to increases in leaf area rather than higher rates of leaf-level photosynthesis at Alptal (Krause and others 2012a). Carbon accumulation in trees occurred despite progressive imbalances in tree mineral nutrition, as indicated by a relative decrease in needle Mg/N by 1.6% per year (Figure 3; McNulty and others 1996; Boxman and others 1998; Minocha and others 2000). Also, N-induced losses of base cations (Figure 1C, D) did not impair tree growth at Alptal. Nitrogen-induced decreases in C/N (stoichiometric N sink; Lovett and Goodale 2011) also contributed to N accumulation at Alptal as N addition decreased C/N of Oe horizon material (Table S4.1), fine roots of the Ah horizon (Table S3.1) and litter (Figure 3C). This is in line with a decrease in soil C/N at Alptal from 1997 to 2009 reported by Schleppi and others (2017). Still, the overall contribution of altered C/N stoichiometry to the N sink was comparatively small and tended to decrease over time (Schleppi and others 2017).

In contrast, changes in C/N stoichiometry appear to dominate ecosystem N retention at Klosterhede. Nitrogen accumulated relative to C in needles, litter (Figure 3D), fine roots (Table S3.2), salt-extractable pools (Table S1.3) and AE soil horizon (Table S4.2). At the same time, trees clearly suffered in N treated plots from 2009 onwards (that is, 17 years after N addition began; Figure 3) in accordance with some results from boreal (Högberg and others 2006) and temperate forests (Magill and others 2004; McNulty and others 2005; Thomas and others 2010; Frey and others 2014). This, in turn, limited the sequestration of added N in new biomass at Klosterhede. Thus, it appears that N addition has saturated the C accumulation component of the vegetation N sink at Klosterhede.

### No Reduction of Fine Root Biomass After N Addition

Nitrogen addition did not affect FRB at Alptal and increased FRB across all horizons at Klosterhede. This was contrary to what we anticipated in our second hypothesis, as FRB typically decreases along natural gradients of N availability (Nadelhoffer and others 1985; Vogt and others 1986; Gundersen and others 1998; Yuan and Chen 2010) and with experimental N addition (Haynes and Gower 1995; Magill and others 2004; Wang and others 2012; Li and others 2015). To reconcile these unexpected results, it is useful to recall that measures of FRB pools integrate fine root production (FRP) and mortality followed by fine root decomposition (FRD).

Neither FRP nor FRD has been measured at the two sites. However, as FRP is fueled by plant C allocated belowground, an upper boundary can be estimated by calculating the total amount of C potentially available for FRP as difference between soil CO_2_ efflux and aboveground litterfall (Raich and Nadelhoffer 1989). This approach showed that estimated total belowground carbon flux (TBCF) was on average lower in N addition plots at both sites (− 28 and − 6% at Alptal and Klosterhede, respectively; Figure S6.2, Tables S6.1 and S6.2), which suggests that less C was available for FRP in N addition plots. This is in line with observations that higher soil N availability can alleviate N limitation of trees, which in turn reduce TBCF and, eventually, FRP (Litton and others 2007; Peng and others 2017). However, inferring FRP from TBCF is subjected to at least two limitations. First, our estimates of TBCF assume that soil, litter and root C pool sizes did not change between treatments over time and that C losses other than due to soil CO_2_ efflux were negligible (Giardina and Ryan 2002). Second, even if TBCF was reduced by N, trees might allocate relatively more C toward FRP at the expense of coarse roots, mycorrhiza and/or root exudates (Giardina and others 2005). Therefore, we cannot exclude higher FRP despite lower estimated TBCF in N addition plots.

However, we deem it more likely that N-induced reductions in TBCF did indeed result in lower FRP at the investigated sites as total BNPP (with FRP as an important component) generally scales with TBCF (Litton and Giardina 2008). An N-induced reduction in FRP thus would require a similar (Alptal) or even larger decrease in FRD (Klosterhede) to explain the observed responses of FRB (Nadelhoffer 2000). Indeed, it has been shown recently that N addition can slow down FRD (Sun and others 2016), which in another experiment contributed 5–51% to observed increases in O horizon SOC pools (Xia and others 2018). Reduced FRD thus offers a likely alternative explanation for the observed increases in FRB pools in response to N. Nevertheless, measurements of FRP and FRD are needed to clarify the involved mechanisms and to estimate the contribution of fine root inputs to SOC pools (Xia and others 2018).

### Shifts in Soil C Pools Were Mainly Driven by Changes in Horizon Thickness and Bulk Density

Soil organic C and STN pools are directly dependent on horizon thickness, bulk density and the elemental concentrations [equation (1)]. However, SOC concentrations only (negatively) responded to N in the Ah horizon of Alptal. Thus, the observed changes in Oe and A horizon SOC and STN pools at the two sites were primarily caused by variations in horizon thickness and bulk density (Tables S4.1, S4.2, S4.3). As we sampled soil based on its pedogenetic horizons, these variations could in part result from our sampling procedure or from intrinsically high spatial heterogeneity rather than from the N addition treatment *per se*. In contrast to a fixed-depth sampling scheme, the sampling of pedogenetic horizon considers soil development and is commonly used for estimating SOC and STN pools (Müller and Kögel-Knabner 2009; Gosheva and others 2017; Poeplau and others 2017). However, to facilitate cross-study comparisons we also calculated: (i) SOC and STN pools per 10-cm increments (Figures 6C, D, 7C, D); and (ii) SOC and STN pools of modeled, 1-cm-thick layers (Tables 2, 3). These corrections either reduce (method i) or eliminate (method ii) the direct influence of horizon thickness on pool sizes while retaining any effects of bulk density.

Calculating SOC pools of modeled, 1-cm-thick layers revealed that direct controls of SOC pools were differently affected by N at the two sites. At Klosterhede, differences in SOC pool sizes were entirely driven by differences in horizon thickness as pools of modeled, 1-cm-thick layers did not differ between treatments (Table 3). For the Oe horizon, this is in line with observations that organic horizon mass often increases with N addition (Mäkipää 1995; Blackwood and others 2007; Pregitzer and others 2008; Zak and others 2008; Lovett and others 2013), which in some studies led to larger forest floor SOC pools (Mäkipää 1995; Olsson and others 2005; Maaroufi and others 2015). Increases in organic horizon thickness (and mass) in response to N can result from larger aboveground litter inputs, reduced decomposition or both. Although N generally slowed down organic matter decomposition in similar experiments (Franklin and others 2003; Burton and others 2004; DeForest and others 2004; Maaroufi and others 2015), evidence for N-induced reductions of Oe horizon decomposition at Klosterhede is unclear. Although N did not significantly alter soil CO_2_ efflux (measured in situ during 2002/2003, Figure S6.2, Table S6.2), potential C mineralization rates per g SOC from Oe material was 1.4-fold higher in N addition plots (Figure S6.1). However, rates of potential C mineralization and soil CO_2_ efflux are not directly comparable as they integrate different sources of CO_2_ and are measured under different conditions.

Estimating Oe horizon turnover by dividing Oe mass (+ 35% with N addition in 2014) by aboveground litter input (+ 11% with N addition on average from 1992 to 2010) showed that organic horizon turnover based on mass balance increased from 22 to 26 years on average with N addition (Figure S6.2, Table S6.2; compare Zak and others 2008 for the mass balance approach). Thus, it appears that both slower decomposition of Oe horizon material and higher litter inputs contributed to the observed increase in Oe horizon thickness at Klosterhede. We suspect, however, that slowed decomposition will be more important for possible future accumulations of Oe material, as higher litter inputs in N addition plots resulted from increased tree mortality rather than from higher NPP.

The N-induced accumulation of SOC in the Oe horizon at Klosterhede was compensated by lower SOC pools due to thinner AE horizons in N addition plots (Tables S4.2, S4.3). Reductions in mineral horizon thickness in response to N are generally not reported in the literature as many studies avoid potential problems with horizon identification by sampling mineral soil in fixed-depth increments (for example, Pregitzer and others 2008). In the long run, however, acidification associated with chronic N addition may enhance the eluviation of organic matter complexed by Al and Fe ions from (A)E horizons of podzols (Funakawa and others 1993; Lundström and others 2000), which is hardly captured with a fixed-depth soil sampling strategy.

At Alptal, Oe horizon SOC pools were still larger in N addition plots after depth normalization (Table 2), emphasizing that increases in bulk density were responsible for the observed increases in SOC pools (Tables S4.1, S4.3). Bulk density itself depends on soil porosity and the density of soil particles (Blume and others 2010). However, if N increased the bulk density of the Oe horizon by reducing pore space, by increasing particle density or by both remains elusive. In the Ah horizon, N-induced decreases in SOC pools sizes were mainly driven by reductions of SOC concentrations (Tables S4.1, S4.3). These could have resulted from decreased inputs via roots or dissolved organic C (DOC) and/or increased outputs via SOC mineralization or DOC leaching. Although DOC concentrations in 5 cm depth were not affected after 3 years of N addition (Hagedorn and others 2001a), we found a trend toward lower fine root C pools, indicating that belowground inputs to the Ah horizon might have been reduced (Figures S3.2, S6.2, Table S6.1). As we found no differences in horizon-specific C output (potential C mineralization rates), these slight reductions in root inputs potentially contributed to lower SOC concentrations in the Ah horizon of N treated plots at Alptal.

## Conclusions

Our results, drawn from two decadelong N addition experiments, indicate that N addition induced a range of responses that were site-specific. Nitrogen enhanced tree growth despite reductions in soil pH and extractable base cations at the pedogenetically ‘younger’ Swiss site. At the pedogenetically ‘older’, nutrient-poor Danish site, however, N addition increased tree mortality and led to N accumulation in several above- and belowground pools. In the soil N addition resulted in vertical shift of SOC pools within the profile that was consistent across sites. Although N induced an accumulation of SOC in the organic layers, there was a concomitant decline in mineral horizon SOC pools. This could have far-reaching implications for long-term C stabilization in temperate forests. Because SOC in the organic layer is subjected to a lower degree of physicochemical protection than in the mineral soil, the former is more likely to be released as CO_2_ in case of disturbances or changing environmental conditions. Thus, a vertical redistribution of SOC from mineral to organic layer pools as a result of increased N deposition may lead to a greater vulnerability of SOC and reduce long-term C sequestration in temperate forest soils.

## Electronic supplementary material

Below is the link to the electronic supplementary material.

Supplementary material 1 (PDF 551 kb)

Supplementary material 2 (XLS 122 kb)

Supplementary material 3 (XLS 177 kb)
