# Supplementary material for: Vertical Redistribution of Soil Organic Carbon Pools After Twenty Years of Nitrogen Addition in Two Temperate Coniferous Forests
Source: Ecosystems. 2018 Jun 26;22(2):379–400. doi: 10.1007/s10021-018-0275-8 (PMC6423314; doi:10.1007/s10021-018-0275-8)
Supplement: Supplementary file 1 — Supplementary material 1 (PDF 551 kb) [file 10021_2018_275_MOESM1_ESM.pdf]

Vertical redistribution of soil organic carbon pools after twenty years of nitrogen addition in two temperate coniferous forests

Stefan J. Forstner[1,*], Viktoria Wechselberger[1], Stefanie Müller[1], Katharina M. Keibinger[1], Eugenio Díaz-Pinés[1], Wolfgang Wanek[2], Patrick Scheppi[3], Frank Hagedorn[3], Per Gundersen[4], Michael Tatzber[1,5], Martin H. Gerzabek[1], Sophie Zechmeister-Boltenstern[1]

[1] Institute of Soil Research, Department of Forest and Soil Sciences, University of Natural Resources and Life Sciences (BOKU), 1190 Vienna, Austria

[2] Department of Microbiology and Ecosystem Science, University of Vienna, 1090 Vienna, Austria

[3] Swiss Federal Institute for Forest, Snow and Landscape Research (WSL), 8903 Birmensdorf, Switzerland

[4] Department of Geosciences and Natural Resource Management, University of Copenhagen, 1958 Frederiksberg C, Denmark

[5] Division of Radiation Protection, Department of Radiation Protection and Radiochemistry, Austrian Agency for Health and Food Safety (AGES), 1220 Vienna, Austria

***Corresponding Author:** stefan.forstner@boku.ac.at; Phone: +43 1 47654-91142

Author contributions S.J.F contributed to study design, performed research, analyzed data and wrote the manuscript; V.W. and S.M. performed research; K.M.K, E.D.-P., F.H. and W.W. co-wrote the manuscript; P.S. and P.G. maintained the N addition experiments, provided data and co-wrote the manuscript; M.T. conceived the study and performed research; M.H.G conceived the study and co-wrote the manuscript; S.Z-B conceived the study, co-wrote the manuscript, was the first supervisor of students and led the project.

Supplementary Material

Appendix S1: Physicochemical soil properties – Supplementary Tables (4)

Appendix S2: Tree growth and N accumulation – Supplementary Table (1)

Appendix S3: Fine roots – Supplementary Figures (3) and Supplementary Tables (2)

Appendix S4: Soil organic C and soil total N pools – Supplementary Text, Supplementary Equations (3) and Supplementary Tables (3)

Appendix S5: Total belowground C and N pools – Supplementary Table (1)

Appendix S6: Potential C mineralization rates and ecosystem C cycling – Supplementary Text, Supplementary Figure (1) and Supplementary Tables (2)

Appendix S7: Statistical considerations regarding the experimental layout at Klosterhede – Supplementary Text and Supplementary Figure (1)

Appendix S1: Physicochemical soil properites

Table S1.1 Gravimetric water content (GWC), soil texture, soil pH, carbonate concentration ($CaCO_3$), cation exchange capacity (CEC_{eff}), base saturation (BS_{eff}), ammonium-N (NH_4^+-N), nitrate-N (NO_3^--N), extractable organic carbon (EOC), extractable organic nitrogen (EON) and EOC:EON (mass ratio) in genetic soil horizons at Alptal. Lower-case letters within columns originate from pairwise comparison of horizon means. Means with no letter in common are significantly different (Tukey's HSD; $\alpha = 0.05$). Bold and bold-italic values indicate significant ($P < 0.05$) and marginally significant ($P < 0.1$) post-hoc differences between treatments within a horizon, respectively. Note that post-hoc differences were found for NH_4^+-N despite a non-significant interaction. Means (±SE) were derived from 4-13 samples per horizon/treatment combination.

	GWC (%)	Sand (%)	Silt (%)	Clay (%)	pH	$CaCO_3$ (%)	CEC_{eff} ($mmol_c$ kg^{-1} soil)	BS_{eff} (%)	NH_4^+-N (µg g^{-1} soil)	NO_3^--N (µg g^{-1} soil)	EOC (µg g^{-1} soil)	EON (µg g^{-1} soil)	EOC:EON (mass ratio)
Oi	a	n.a.	n.a.	n.a.	*ab*	n.a.	n.a.	n.a.	*a*	*a*	*a*	*a*	*ab*
Control	66.7 ± 2.4				6.0 ± 0.1				410.8 ± 49.7	**1.1 ± 0.4**	5614.9 ± 1032.6	687.8 ± 63.9	7.6 ± 0.9
+ Nitrogen	66.9 ± 4.0				5.7 ± 0.2				465.2 ± 71.0	**9.7 ± 3.8**	3376.0 ± 449.7	587.5 ± 54.2	6.0 ± 0.7
Oe	ab	n.a.	n.a.	n.a.	*ac*	n.a.	n.a.	n.a.	*a*	*a*	*b*	*b*	*ac*
Control	74.5 ± 2.6				5.6 ± 0.3				**468.1 ± 158.6**	1.9 ± 0.8	2539.3 ± 377.3	465.7 ± 61.9	**5.7 ± 0.6**
+ Nitrogen	74.9 ± 2.9				5.2 ± 0.2				**730.2 ± 113.7**	5.1 ± 2.8	1568.1 ± 127.2	476.2 ± 59.1	**3.6 ± 0.4**
Oa	b	n.a.	n.a.	n.a.	*c*	n.a.	n.a.	n.a.	*b*	*b*	*b*	*b*	*c*
Control	78.7 ± 1.8				5.5 ± 0.3				172.6 ± 14.7	***n.d.***	1669.4 ± 169.0	368.3 ± 22.4	**4.5 ± 0.3**
+ Nitrogen	75.0 ± 3.4				4.6 ± 0.1				239.7 ± 52.3	***2.6 ± 2.0***	1207.4 ± 88.3	417.9 ± 50.7	**3.0 ± 0.3**
Ah	a	*a*	*a*		*c*				*c*	*a*	*c*	*c*	*b*
Control	***69.3 ± 2.6***	2.6 ± 1.3	38.1 ± 8.8	59.3 ± 10.0	5.3 ± 0.2	0.5 ± 0.3	559.6 ± 136.9	93.6 ± 5.2	23.5 ± 7.0	9.2 ± 3.2	463.6 ± 51.8	73.7 ± 10.9	6.7 ± 0.4
+ Nitrogen	***64.3 ± 2.6***	1.6 ± 0.4	35.5 ± 0.9	62.9 ± 1.1	5.1 ± 0.2	0.3 ± 0.1	292.4 ± 115.5	89.8 ± 6.0	37.2 ± 9.7	9.4 ± 4.8	412.0 ± 31.2	78.1 ± 8.0	5.6 ± 0.4
Bl	c	*a*	*a*		*b*				*d*	*a*	*d*	*d*	*ab*
Control	47.8 ± 2.0	2.3 ± 0.9	39.2 ± 2.9	58.5 ± 3.7	6.2 ± 0.2	0.3 ± 0.1	265.5 ± 36.9	97.4 ± 1.8	4.5 ± 0.8	4.3 ± 1.4	117.4 ± 17.7	20.9 ± 2.4	5.5 ± 0.4
+ Nitrogen	48.0 ± 2.7	2.0 ± 0.5	38.1 ± 2.8	60.0 ± 3.0	5.8 ± 0.2	0.3 ± 0.1	196.2 ± 57.9	69.9 ± 13.1	5.9 ± 2.1	2.6 ± 1.3	136.6 ± 14.1	22.3 ± 2.3	6.5 ± 0.7
Blr[a]	c	*a*	*a*		*d*				*d*	*ab*	*e*	*e*	*b*
Control	43.6 ± 1.3	2.0 ± 0.6	39.0 ± 0.9	59.0 ± 0.9	6.6 ± 0.2	0.6 ± 0.4	349.4 ± 8.9	99.9 ± 0.1	4.1 ± 0.7	0.6 ± 0.2	74.3 ± 11.5	**11.9 ± 1.1**	**6.5 ± 1.1**
+ Nitrogen	43.1 ± 1.3	1.6 ± 0.4	41.0 ± 1.8	57.4 ± 1.9	6.6 ± 0.2	0.7 ± 0.4	242.6 ± 39.6	99.3 ± 0.4	2.6 ± 0.5	1.0 ± 0.4	62.6 ± 13.2	**3.7 ± 0.9**	**28.9 ± 14.0**
Sign. effects	Horizon				+N, Horizon		+N		Horizon	Horizon, +N x Horizon	+N, Horizon	+*N*[b], Horizon, +N x Horizon	Horizon, +N x Horizon

n.a. = not analyzed.

[a] Blr horizons were sampled to an average depth of 30.0±2.1 cm and 26.3±1.0 cm from top of mineral soil in control and +N treatment plots, respectively.

[b] Marginally significant main effect (P=0.089).

Table S1.2 Exchangeable cations in mineral soil horizons at Alptal. Lower-case letters within columns originate from pairwise comparison of horizon means. Means with no letter in common are significantly different (Tukey's HSD; α = 0.05). Means (±SE) were derived from 4 samples per horizon/treatment combination.

	Na^+	K^+	Mg^{2+}	Ca^{2+}	Base cations ($mmol_c\ kg^{-1}$ soil)	Al^{3+}	Mn^{2+}	Fe^{3+}	Acidic cations
Ah			*a*	*a*		*ab*			*a*
Control	0.54 ± 0.05	3.34 ± 0.85	17.54 ± 2.97	520.63 ± 145.94	542.05 ± 147.46	13.45 ± 9.73	1.33 ± 0.43	2.80 ± 1.68	17.57 ± 11.56
+ Nitrogen	0.45 ± 0.06	2.73 ± 0.44	13.21 ± 2.91	256.91 ± 118.57	273.31 ± 121.16	17.97 ± 10.89	0.93 ± 0.54	0.17 ± 0.14	19.07 ± 10.77
Bl			*a*	*a*		*a*			*a*
Control	0.46 ± 0.03	2.46 ± 0.17	10.87 ± 0.50	246.04 ± 38.98	259.82 ± 39.37	5.07 ± 3.77	0.34 ± 0.10	0.28 ± 0.25	5.68 ± 3.92
+ Nitrogen	0.55 ± 0.12	2.68 ± 0.37	10.05 ± 1.42	140.73 ± 70.79	154.01 ± 71.98	40.53 ± 17.51	0.49 ± 0.16	1.22 ± 0.68	42.24 ± 18.11
Blr[a]			*a*	*a*		*b*			*b*
Control	0.49 ± 0.05	2.39 ± 0.13	9.66 ± 2.16	336.61 ± 6.58	349.14 ± 8.64	0.02 ± 0.02	0.28 ± 0.21	0.00 ± 0.00	0.29 ± 0.23
+ Nitrogen	0.43 ± 0.05	2.24 ± 0.15	11.16 ± 0.61	227.41 ± 40.00	241.24 ± 40.10	0.21 ± 0.12	0.56 ± 0.22	0.58 ± 0.35	1.34 ± 0.69
Sign. effects			*Horizon*[b]	+N, *Horizon*[c]	+N, *Horizon*[d]	Horizon			Horizon

[a] Blr horizons were sampled to an average depth of 30.0±2.1 cm and 26.3±1.0 cm from top of mineral soil in control and N addition plots, respectively.
Marginally significant main effects ([b]P=0.086,[c]P=0.068,[d]P=0.070).

Table S1.3 Gravimetric water content (GWC), soil texture, soil pH, carbonate concentration ($CaCO_3$), cation exchange capacity (CEC_{eff}), base saturation (BS_{eff}), ammonium-N (NH_4^+-N), nitrate-N (NO_3^--N), extractable organic carbon (EOC), extractable organic nitrogen (EON) and EOC:EON (mass ratio) in genetic soil horizons at Klosterhede. Lower-case letters within columns originate from pairwise comparison of horizon means. Means with no letter in common are significantly different (Tukey's HSD; $\alpha = 0.05$). Bold and bold-italic values indicate significant ($P < 0.05$) and marginally significant ($P < 0.1$) post-hoc differences between treatments within a horizon, respectively. Note that post-hoc differences were found for soil pH despite a non-significant interaction. Means (±SE) were derived from 3-12 samples per horizon/treatment combination.

	GWC (%)	Sand (%)	Silt (%)	Clay (%)	pH	CEC_{eff} ($mmol_c$ kg^{-1} soil)	BS_{eff} (%)	NH_4^+-N (µg g^{-1} soil)	NO_3^--N (µg g^{-1} soil)	EOC (µg g^{-1} soil)	EON (µg g $soil^{-1}$)	EOC:EON (mass ratio)
Oe	*a*	n.a.	n.a.	n.a.	*a*	n.a.	n.a.	*a*†	*a*†	*a*	*a*†	*a*
Control	69.0 ± 0.5				4.4 ± 0.0			230.3 ± 18.4	**n.d.**	1221.3 ± 85.4	263.2 ± 18.9	**4.7 ± 0.3**
+ Nitrogen	67.4 ± 0.9				4.5 ± 0.0			425.4 ± 25.0	**1.4 ± 0.6**	1236.6 ± 101.0	456.8 ± 27.2	**2.7 ± 0.2**
Oa	*a*	n.a.	n.a.	n.a.	*b*	n.a.	n.a.	*b*†	*b*†	*b*	*b*†	*a*
Control	64.8 ± 1.0				4.3 ± 0.0			117.4 ± 9.6	**n.d.**	686.1 ± 44.9	149.1 ± 9.7	**4.7 ± 0.3**
+ Nitrogen	62.1 ± 2.6				4.2 ± 0.0			172.8 ± 14.3	**4.1 ± 1.0**	698.0 ± 48.2	198.9 ± 12.1	**3.7 ± 0.3**
AE	*b*	*a*	*a*	*a*	*a*	*a*	*a*	*a*‡	*a*‡	*c*	*ab*‡	*b*
Control	15.6 ± 0.7	77.7 ± 3.6	17.0 ± 2.9	5.3 ± 0.7	4.5 ± 0.0	5.3 ± 1.0	58.3 ± 8.6	3.6 ± 0.5	0.1 ± 0.0	84.8 ± 6.8	**10.2 ± 0.6**	**8.4 ± 0.6**
+ Nitrogen	17.2 ± 1.1	73.4 ± 1.5	19.7 ± 0.5	6.8 ± 1.3	4.5 ± 0.1	6.2 ± 1.7	61.6 ± 3.9	6.9 ± 1.0	0.4 ± 0.1	90.1 ± 7.1	**15.1 ± 1.2**	**6.2 ± 0.5**
E	*c*	*a*	*a*	*a*	*ac*	*a*	*b*	*b*‡	*b*‡	*c*	*a*‡	*b*
Control	12.0 ± 0.5	77.7 ± 0.5	15.5 ± 0.7	6.8 ± 0.2	***4.6 ± 0.0***	5.1 ± 1.0	33.4 ± 4.0	2.5 ± 0.5	n.d.	77.4 ± 11.5	**10.3 ± 1.4**	8.0 ± 1.1
+ Nitrogen	12.4 ± 0.5	78.3 ± 1.2	15.3 ± 1.4	6.3 ± 0.6	***4.5 ± 0.1***	4.4 ± 0.6	29.7 ± 6.6	4.5 ± 0.5	0.2 ± 0.1	83.6 ± 8.1	**13.1 ± 1.1**	6.7 ± 0.6
Bh	*d*	*b*	*a*	*b*	*a*	*b*	*b*	*b*‡	*b*‡	*d*	*c*‡	*c*
Control	25.3 ± 1.0	62.3 ± 1.9	21.1 ± 2.9	16.7 ± 1.4	4.5 ± 0.1	12.2 ± 1.0	25.8 ± 1.0	1.7 ± 0.4	n.d.	379.3 ± 25.3	15.1 ± 0.8	25.6 ± 1.6
+ Nitrogen	24.9 ± 0.7	64.2 ± 1.3	18.1 ± 1.0	17.7 ± 1.6	4.6 ± 0.0	10.5 ± 1.2	23.7 ± 2.4	5.4 ± 1.1	0.2 ± 0.1	353.7 ± 20.7	17.0 ± 1.6	21.9 ± 1.5
Bs [a]	*e*	*b*	*a*	*b*	*c*	*c*	*b*	*b*‡	*a*‡	*e*	*bc*‡	*c*
Control	**20.2 ± 0.7**	66.8 ± 2.1	21.9 ± 2.0	11.3 ± 0.7	4.6 ± 0.0	2.5 ± 0.1	28.6 ± 2.2	2.6 ± 0.4	0.2 ± 0.1	291.0 ± 27.6	13.2 ± 0.7	21.7 ± 1.3
+ Nitrogen	**17.9 ± 0.4**	71.5 ± 2.2	18.7 ± 1.5	9.8 ± 0.7	4.7 ± 0.1	2.6 ± 0.2	24.8 ± 1.8	3.4 ± 0.4	0.4 ± 0.1	245.0 ± 20.3	13.8 ± 0.6	17.7 ± 1.2
Sign. effects	Horizon	Horizon	*Horizon*[b]	Horizon	Horizon	Horizon	Horizon	Org: +N, Horizon<br>Min: +N, Horizon	Org: +N, Horizon, +N x Horizon<br>Min: +N, Horizon	Horizon	Org: +N, Horizon<br>Min: +N, Horizon, *+N x Horizon*[c]	+N, Horizon, +N x Horizon

n.d. = not detected; n.a. = not analyzed.
[a] Bs horizons were sampled to an average depth of 31.6±1.3 cm and 29.7±0.8 cm from top of mineral soil in control and +N treatment plots, respectively.
Marginally significant main effect and treatment x horizon interactions ([b]P=0.071,[c]P=0.097,[d]P=0.099).
† Lower-case letters indicate significant post-hoc differences between organic horizons.
‡ Lower-case letters indicate significant post-hoc differences between mineral horizons.

Table S1.4 Exchangeable cations in mineral soil horizons at Klosterhede. Lower-case letters within columns originate from pairwise comparison of horizon means. Means with no letter in common are significantly different (Tukey's HSD; α = 0.05). Means (±SE) were derived from 4 samples per horizon/treatment combination.

	Na^+	K^+	Mg^{2+}	Ca^{2+}	Base cations ($mmol_c$ kg^{-1} soil)	Al^{3+}	Mn^{2+}	Fe^{3+}	Acidic cations
AE	*ab*	*a*	*a*	*a*	*a*	*a*		*a*	*a*
Control	1.00 ± 0.38	0.29 ± 0.05	1.83 ± 0.61	0.19 ± 0.09	3.31 ± 1.12	0.59 ± 0.05	n.d.	1.40 ± 0.24	1.99 ± 0.24
+ Nitrogen	1.12 ± 0.37	0.33 ± 0.07	2.26 ± 0.77	0.23 ± 0.09	3.94 ± 1.30	0.81 ± 0.17	n.d.	1.42 ± 0.28	2.23 ± 0.44
E	*ac*	*b*	*b*	*a*	*b*	*b*		*a*	*b*
Control	0.58 ± 0.10	0.19 ± 0.02	0.85 ± 0.20	0.11 ± 0.03	1.73 ± 0.35	1.94 ± 0.32	n.d.	1.48 ± 0.34	3.42 ± 0.66
+ Nitrogen	0.48 ± 0.09	0.21 ± 0.02	0.46 ± 0.10	0.09 ± 0.02	1.24 ± 0.22	2.05 ± 0.42	n.d.	1.16 ± 0.24	3.20 ± 0.66
Bh	*b*	*a*	*a*	*a*	*a*	*c*		*b*	*c*
Control	1.28 ± 0.16	0.36 ± 0.01	1.40 ± 0.24	0.13 ± 0.02	3.17 ± 0.37	6.56 ± 0.31	n.d.	2.48 ± 0.37	9.04 ± 0.66
+ Nitrogen	0.92 ± 0.11	0.34 ± 0.03	1.03 ± 0.21	0.15 ± 0.03	2.44 ± 0.30	5.91 ± 0.65	n.d.	2.10 ± 0.49	8.01 ± 0.99
Bs[a]	*c*	*b*	*c*	*b*	*c*	*b*		*c*	*a*
Control	0.45 ± 0.08	0.12 ± 0.02	0.13 ± 0.01	0.03 ± 0.02	0.72 ± 0.07	1.43 ± 0.06	n.d.	0.35 ± 0.04	1.78 ± 0.06
+ Nitrogen	0.36 ± 0.06	0.12 ± 0.02	0.14 ± 0.03	0.03 ± 0.00	0.65 ± 0.09	1.56 ± 0.11	n.d.	0.39 ± 0.06	1.95 ± 0.17
Sign. effects	Horizon	Horizon	Horizon	Horizon		Horizon		Horizon	

[a] Bs horizons were sampled to an average depth of 31.6±1.3 cm and 29.7±0.8 cm from top of mineral soil in control and +N treatment plots, respectively.

Appendix S2: Tree growth and N accumulation

Table S2.1 Linear regression parameters of selected aboveground and belowground responses to N addition as depicted in Figure 3 of the main text.

Equation	df	P(Slope)	RSE	R^2	Adj. R^2
Panel (a) – Alptal					
$BAI = 1.15 \times Year - 2219.53$	12	<0.001	3.16	0.71	0.69
$Needle\,biomass = 1.16 \times Year - 2199.22$	13	0.048	8.85	0.27	0.21
Panel (b) – Klosterhede					
$BAI = -0.84 \times Year + 1765.98$	19	0.262	22.29	0.07	0.02
† $BAI = -1.59 \times Year + 3231.02$	18	0.023	18.14	0.26	0.21
Panel (c) – Alptal					
$Needle\,Mg:N = -1.61 \times Year + 3300.07$	9	0.015	5.61	0.50	0.45
$Litter\,N = 1.67 \times Year - 3237.4$	12	0.002	6.49	0.56	0.52
Panel (d) – Klosterhede					
¶ $Needle\,N\,conc. = 1.08 \times Year - 2034.90$	4	0.041	5.40	0.69	0.61
¶ $Needle\,Mg:N = -1.23 \times Year + 2536.86$	4	0.111	9.08	0.51	0.39
$Litter\,N = 1.86 \times Year - 3600.0$	3	0.004	3.39	0.96	0.94

RSE stands for residual standard error, BAI stands for basal area increment.
† Linear regression excluding data from 1992.
¶ Linear regression excluding data from 1999 (no N was applied in 1998-1999).

Appendix S3: Fine roots

Figure S3.1 Fine root mass (<2 mm diameter), coarse root mass (>2 mm diameter) and particulate organic matter mass (POM) in genetic soil horizons at Alptal. Roots that could not be assigned to a specific organic horizon were measured separately (‘Org’). Particulate organic matter was not determined for these horizons (‘Org’). Symbols between bars indicate significant post-hoc differences for a given fraction between treatments within a given horizon (■$P < 0.1$,*$P < 0.05$, **$P < 0.01$, ***$P < 0.001$). Means (±SE) were derived from 6-13 samples per horizon/treatment combination.

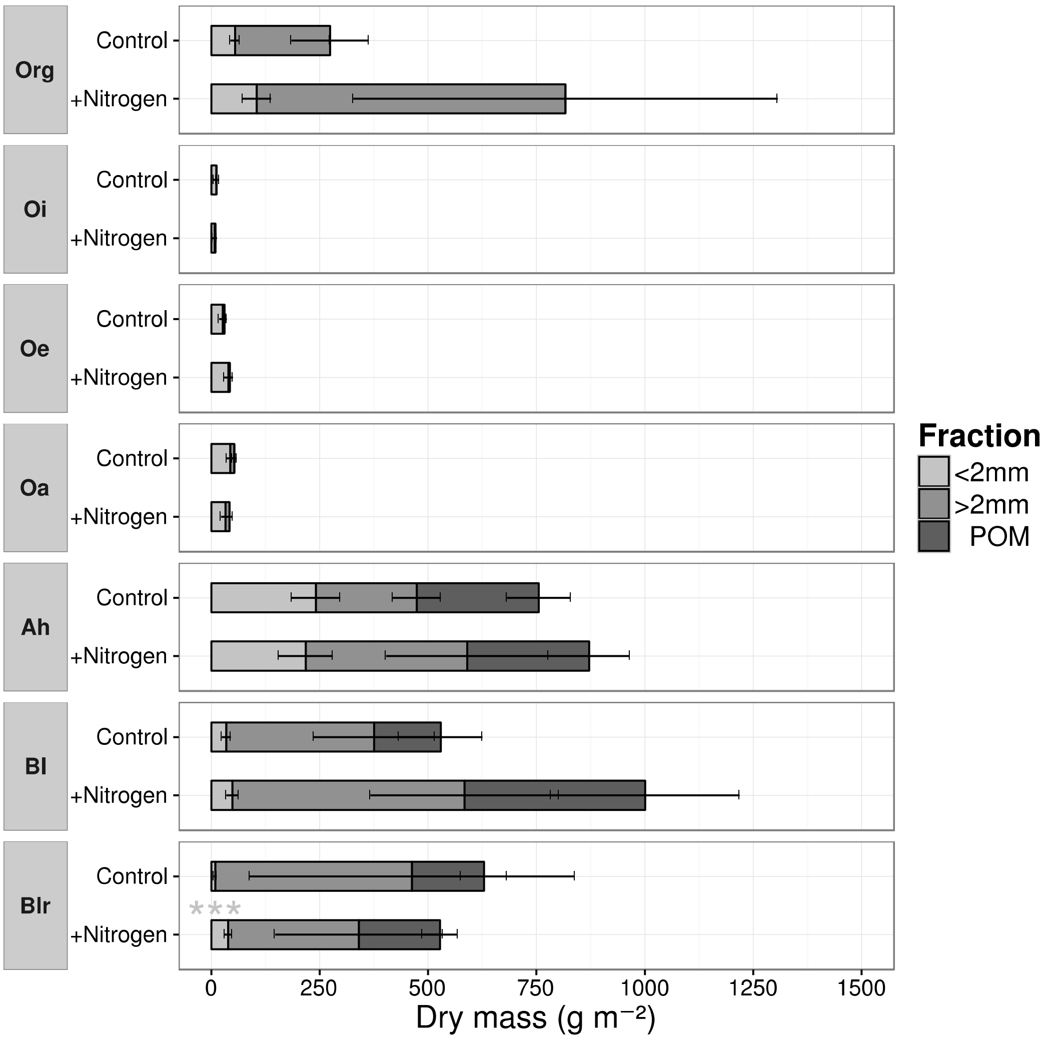

Figure S3.2 Fine root C pools (a,c) and fine root N pools (b,d) in genetic soil horizons (upper panels) and depth increments (lower panels) at Alptal. Lower-case letters right to each plate originate from pairwise comparison of horizon/increment means. Means with no letter in common are significantly different (Tukey's HSD; α = 0.05). Means (±SE) were derived from 12 samples per horizon/treatment combination.

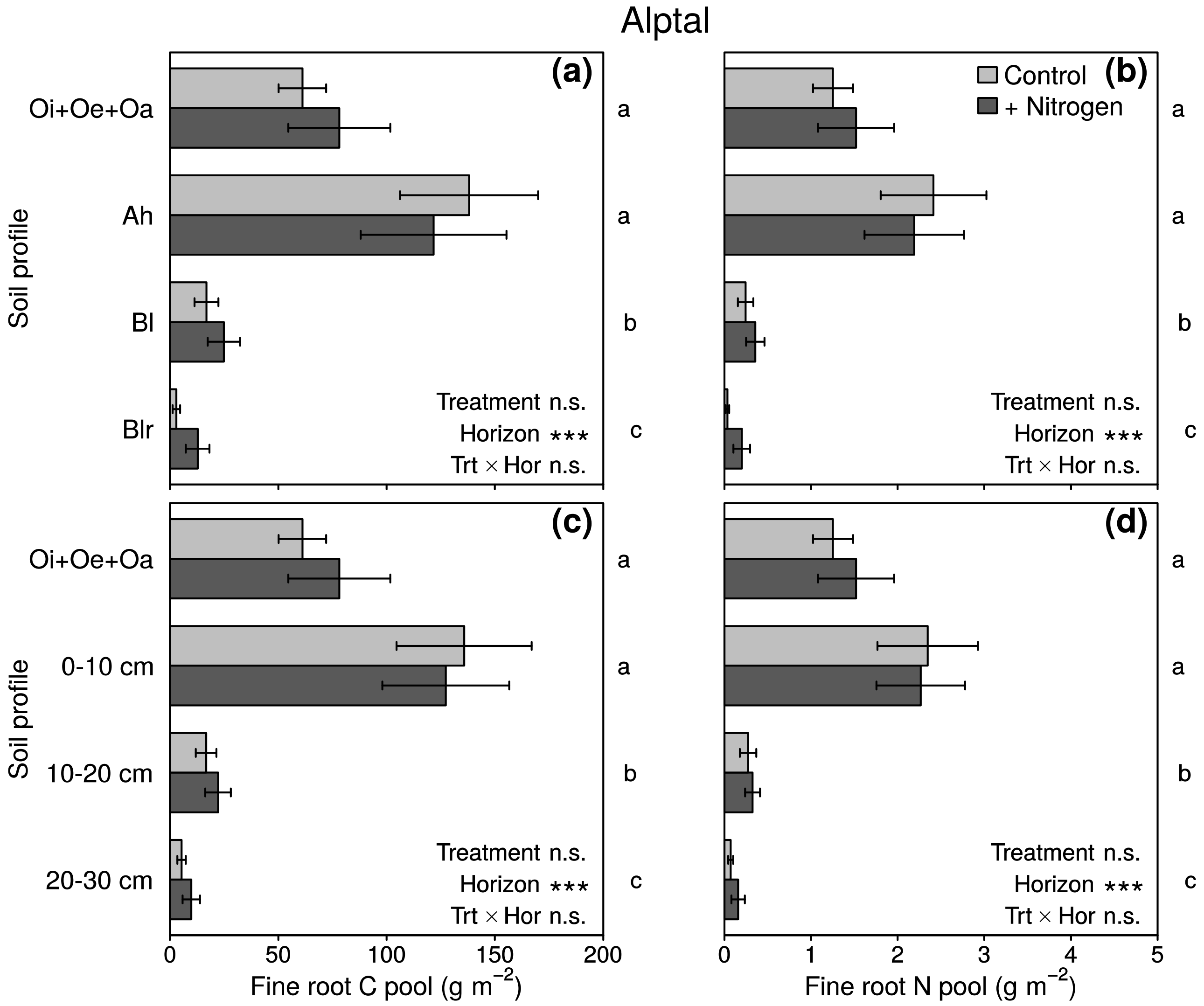

Figure S3.3 Fine root mass (<2mm diameter), coarse root mass (>2mm diameter) and particulate organic matter mass (POM) in genetic soil horizons at Klosterhede. Roots that could not be assigned to a specific organic horizon were measured separately (‘Org’). Particulate organic matter was not determined for these horizons (‘Org’). Symbols between bars indicate significant post-hoc differences for a given fraction between treatments within a given horizon (▪$P < 0.1$,*$P < 0.05$, **$P < 0.01$, ***$P < 0.001$). The N addition main effects for fine root mass and coarse root mass are significant at $P = 0.016$ and $P = 0.060$, respectively. Means (±SE) were derived from 9-12 samples per horizon/treatment combination.

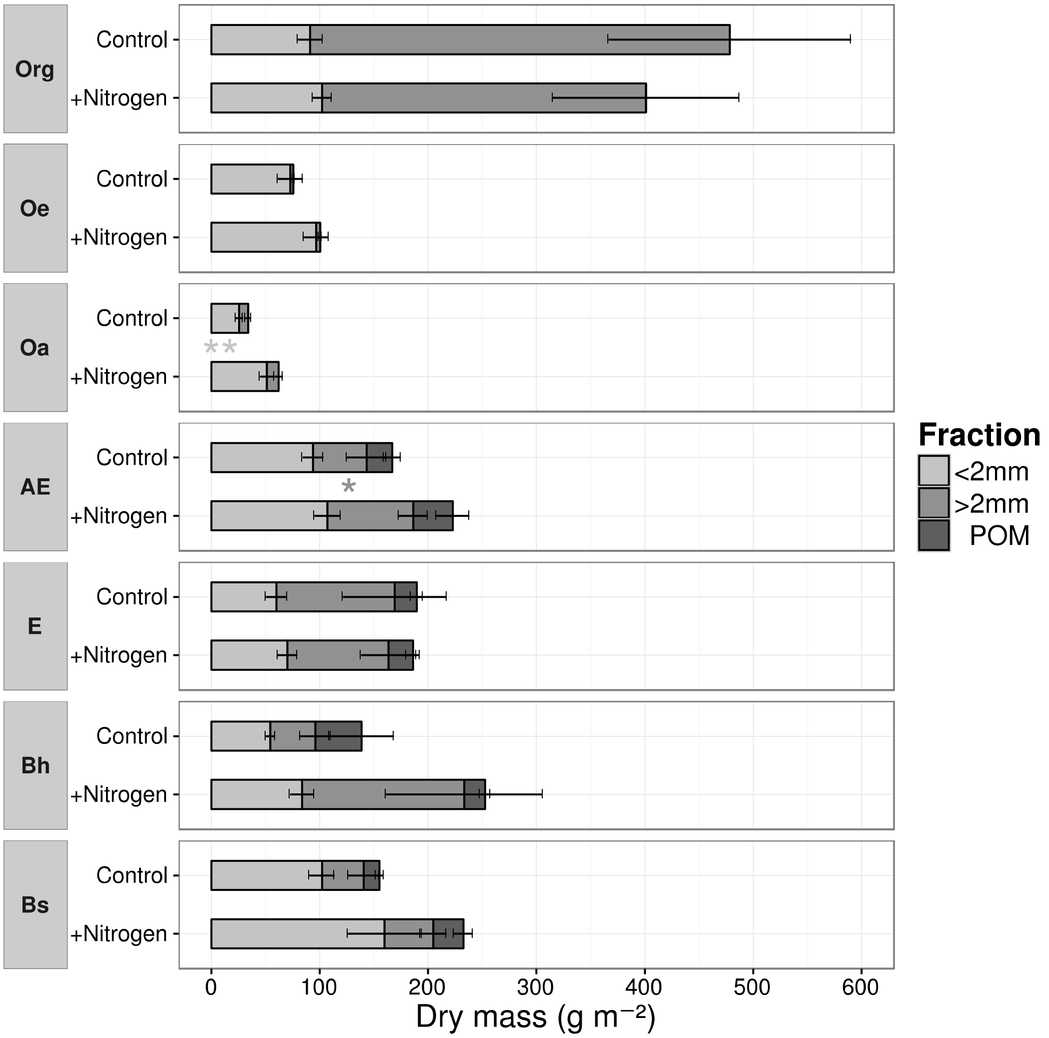

Table S3.1 Fine root C (FRC) concentrations, fine root N (FRN) concentrations and FRC:FRN (mass ratio) in genetic soil horizons at Alptal. Roots that could not be assigned to a specific organic horizon were measured separately (‘Org’). Lower-case letters within columns originate from pairwise comparison of horizon means. Means with no letter in common are significantly different (Tukey’s HSD; $\alpha = 0.05$). Bold and bold-italic values indicate significant ($P < 0.05$) and marginally significant ($P < 0.1$) post-hoc differences between treatments within a horizon, respectively. Note that post-hoc differences were found for FRC concentrations in the Bl horizon, as well as for FRN concentrations and FRC:FRN in the Ah horizon, despite non-significant interactions. Means (±SE) were derived from 3-13 samples per horizon/treatment combination.

	Fine root C (mass%)	Fine root N (mass%)	FRC:FRN (mass ratio)	n
Org				
Control	53.60 ± 0.57	0.95 ± 0.06	59.09 ± 4.05	11
+ Nitrogen	54.26 ± 0.37	1.01 ± 0.08	57.16 ± 4.56	11
Oi		*a*	*a*	
Control	49.87 ± 0.33	1.40 ± 0.11	36.60 ± 3.43	5
+ Nitrogen	50.74 ± 1.52	1.50 ± 0.17	35.54 ± 4.87	4
Oe		*a*	*a*	
Control	51.01 ± 0.55	1.34 ± 0.13	40.67 ± 4.29	7
+ Nitrogen	51.89 ± 0.27	1.36 ± 0.06	38.83 ± 2.34	8
Oa		*a*	*a*	
Control	50.80 ± 0.48	1.14 ± 0.06	45.84 ± 2.24	11
+ Nitrogen	52.38 ± 0.30	1.14 ± 0.07	46.92 ± 2.83	6
Ah		*b*	*b*	
Control	51.89 ± 0.71	**0.83 ± 0.05**	**65.30 ± 4.08**	13
+ Nitrogen	51.20 ± 0.65	**1.04 ± 0.07**	**51.55 ± 3.22**	13
Bl		*c*	*c*	
Control	***50.07 ± 0.71***	0.70 ± 0.05	74.76 ± 6.79	9
+ Nitrogen	***51.64 ± 0.41***	0.71 ± 0.07	84.61 ± 17.07	8
Blr[a]		*bc*	*bc*	
Control	52.32 ± 0.47	0.68 ± 0.14	85.18 ± 19.06	3
+ Nitrogen	51.02 ± 0.74	0.76 ± 0.10	70.80 ± 6.64	5
Sign. effects		Horizon	Horizon	

[a] Blr horizons were sampled to an average depth of 30.0±2.1 cm and 26.3±1.0 cm from top of mineral soil in control and +N treatment plots, respectively.

Table S3.2 Fine root C (FRC) concentrations, fine root N (FRN) concentrations and FRC:FRN (mass ratio) in genetic soil horizons at Klosterhede. Roots that could not be assigned to a specific organic horizon have been measured separately ('Org'). Lower-case letters within columns originate from pairwise comparison of horizon means. Means with no letter in common are significantly different (Tukey's HSD; α = 0.05). Bold values indicate significant N addition effects for 'Org' roots ($P < 0.05$). Bold-italic values indicate marginally significant ($P < 0.1$) post-hoc differences between treatments within a horizon. Note that post-hoc differences were found for FRC concentrations in the Bs horizon despite a non-significant interaction. Means (±SE) were derived from 9-12 samples.

	Fine root C (mass%)	Fine root N (mass%)	FRC:FRN (mass ratio)	n
Org				
Control	47.62 ± 0.09	**0.71 ± 0.02**	**67.57 ± 1.90**	12
+ Nitrogen	47.70 ± 0.09	**1.04 ± 0.02**	**46.15 ± 0.88**	12
Oe	*a*	*a*	*a*	
Control	47.45 ± 0.12	1.01 ± 0.02	47.07 ± 1.04	12
+ Nitrogen	47.39 ± 0.08	1.35 ± 0.02	35.31 ± 0.50	12
Oa	*b*	*b*	*b*	
Control	46.70 ± 0.12	0.91 ± 0.02	51.63 ± 1.41	12
+ Nitrogen	46.59 ± 0.11	1.09 ± 0.03	43.27 ± 1.33	10
AE	*b*	*b*	*bc*	
Control	46.55 ± 0.21	0.81 ± 0.02	57.89 ± 1.93	12
+ Nitrogen	46.24 ± 0.22	1.11 ± 0.04	42.23 ± 1.48	12
E	*c*	*c*	*cd*	
Control	43.52 ± 1.08	0.71 ± 0.03	62.48 ± 3.29	11
+ Nitrogen	43.56 ± 0.87	0.99 ± 0.04	44.87 ± 2.12	12
Bh	*d*	*c*	*d*	
Control	42.79 ± 0.45	0.69 ± 0.02	62.11 ± 1.93	10
+ Nitrogen	42.49 ± 0.57	0.90 ± 0.04	48.29 ± 2.67	9
Bs[a]	*d*	*d*	*e*	
Control	***42.80 ± 0.29***	0.54 ± 0.02	80.12 ± 3.18	9
+ Nitrogen	***40.09 ± 1.28***	0.71 ± 0.04	57.53 ± 1.67	11
Sign. effects	Horizon	+N, Horizon, *+N x Horizon*[b]	+N, Horizon	

[a] Bs horizons were sampled to an average depth of 31.6±1.3 cm and 29.7±0.8 cm from top of mineral soil in control and +N treatment plots, respectively.
[b] Marginally significant interaction (P=0.087).

Appendix S4: Soil organic C and soil total N pools

For estimation of SOC and STN pools (Equation 1 in the main text), we calculated bulk density ($\rho_{B,i}$) as

$$\rho_{B,i} = \frac{M_i}{V_i} \tag{S1}$$

where M_i is dry mass (kg) and V_i is the volume (m^3) of root-free (for organic horizons) or 2mm-sieved (for mineral horizons) soil material, respectively. Soil volume V_i (m^3) was calculated for horizon *i* by correcting the nominal sampling volume $V_{N,i}$ for volume of roots and stones as

$$V_i = V_{N,i} - V_{R,i} - V_{S,i} = \left(A_{N,i} \times d_i\right) - FM_{R,i} - \left(\frac{M_{S,i}}{2.65}\right) \tag{S2}$$

where $V_{N,i}$ is the nominal sampling volume of horizon *i* (m^{-3}), $V_{R,i}$ is root volume (m^{-3}) and $V_{S,i}$ is stone volume (m^{-3}). The nominal sampling volume was calculated from nominal sampling area $A_{N,i}$ (0.0625 m^2 for organic horizons, 0.0064 m^2 for mineral horizons) and horizon thickness d_i (m). Root volume was estimated by root fresh mass ($FM_{R,i}$, Mg) assuming a root density of 1 Mg m^{-3} (Birouste and others 2014). Stone volume was calculated as stone dry mass ($M_{S,i}$, Mg) divided by the density of quartz (2.65 Mg m^{-3}). Similarly, the volume fraction of roots and stones θ_i used in Equation 1 of the main text was calculated as

$$\theta_i = \frac{V_{R,i} + V_{S,i}}{V_{N,i}} \tag{S3}$$

The mean volume fraction of roots and stones across all horizons ranged from 0.02 to 0.04 at Klosterhede and from 0.03 to 0.06 at Alptal.

Although we used a corer of 30 cm length, the lower boundaries of the lowest horizons (Blr at Alptal and Bs at Klosterhede) measured on the retrieved cores slightly departed from this value. We therefore determined the actual lower boundaries of these horizons on the retrieved cores in the field. We then used these values to determine horizon thickness d_i for calculations of the nominal soil volume

(Equation S2), bulk density (Equation S1) and volume fraction of roots and stones (Equation S3). For the calculation of SOC/STN pool sizes (Equation 1 in the main text), however, we mathematically normalized the lower horizon boundaries of to 30 cm before calculating d_i to control for the slight variation in sampling depths. In other words, pool sizes for Blr and Bs horizons were calculated using measured values for bulk density and volume fraction of roots and stones, but lower horizon boundaries were truncated or extended to exactly match 30 cm before Blr/Bs thickness was calculated.

Table S4.1 Horizon thickness, soil dry mass, bulk density (ρ_B), soil organic carbon (SOC) concentrations, soil total nitrogen (STN) concentrations and SOC:STN (mass ratio) in genetic soil horizons at Alptal. Lower-case letters within columns originate from pairwise comparison of horizon means. Means with no letter in common are significantly different (Tukey's HSD; $\alpha = 0.05$). Bold and bold-italic values indicate significant ($P < 0.05$) and marginally significant ($P < 0.1$) post-hoc differences between treatments within a horizon, respectively. Note that post-hoc differences were found for SOC, STN, and SOC:STN despite non-significant interactions. Means (±SE) were derived from 6-13 samples per horizon/treatment combination.

	Thickness (cm)	Soil mass (kg m^{-2})	ρ_B (g cm^{-3})	SOC (mg g^{-1} soil)	STN (mg g^{-1} soil)	SOC:STN (mass ratio)
Oi	*a*	*a*	*a*	*a*	*a*	*a*
Control	1.2 ± 0.2	0.6 ± 0.1	0.06 ± 0.01	470.7 ± 14.6	13.1 ± 0.4	36.6 ± 2.0
+ Nitrogen	1.3 ± 0.3	0.9 ± 0.2	0.08 ± 0.02	495.8 ± 13.1	15.5 ± 1.0	33.1 ± 2.0
Oe	*ab*	*ab*	*a*	*ab*	*b*	*b*
Control	1.6 ± 0.2	**0.9 ± 0.2**	**0.05 ± 0.01**	514.0 ± 20.0	19.0 ± 1.1	**27.3 ± 0.8**
+ Nitrogen	2.4 ± 0.3	**2.2 ± 0.6**	**0.09 ± 0.01**	458.0 ± 31.3	20.5 ± 2.1	**23.1 ± 1.5**
Oa	*b*	*b*	*a*	*b*	*ab*	*b*
Control	3.5 ± 0.8	***1.8 ± 0.6***	**0.06 ± 0.01**	399.6 ± 20.3	17.2 ± 1.0	23.6 ± 0.9
+ Nitrogen	2.7 ± 0.4	***2.7 ± 0.5***	**0.11 ± 0.01**	382.1 ± 25.1	16.0 ± 1.5	24.5 ± 2.1
Ah	*c*	*c*	*b*	*c*	*c*	*c*
Control	8.0 ± 0.7	**22.8 ± 3.7**	0.30 ± 0.03	**221.2 ± 31.6**	***10.3 ± 1.4***	21.3 ± 0.8
+ Nitrogen	6.5 ± 0.9	**15.5 ± 3.5**	0.25 ± 0.03	**169.0 ± 17.2**	***8.3 ± 0.8***	20.3 ± 0.5
Bl	*c*	*d*	*c*	*d*	*d*	*c*
Control	9.5 ± 1.1	68.5 ± 11.2	0.74 ± 0.07	44.2 ± 6.0	2.4 ± 0.3	18.5 ± 0.6
+ Nitrogen	9.1 ± 0.8	54.4 ± 6.3	0.63 ± 0.07	55.8 ± 6.5	2.9 ± 0.3	19.2 ± 0.4
Blr[a]	*d*	*d*	*c*	*d*	*e*	*c*
Control	11.5 ± 1.5	95.9 ± 8.2	0.93 ± 0.05	36.3 ± 4.2	1.8 ± 0.2	20.4 ± 0.8
+ Nitrogen	13.9 ± 0.9	80.8 ± 10.0	0.82 ± 0.07	32.1 ± 2.5	1.7 ± 0.1	19.5 ± 1.0
Sign. effects	Horizon	Horizon, +N x Horizon	Horizon, +N x Horizon	Horizon	Horizon	Horizon

[a] Lower horizon boundary of Blr horizons was normalized to 30 cm for calculation of horizon thickness. Soil mass and bulk density were calculated using original sampling depths of 30.0±2.1 cm and 26.3±1.0 cm from top of mineral soil in control and +N plots, respectively.

Table S4.2 Horizon thickness, soil dry mass, bulk density (ρ_B), soil organic carbon (SOC) concentrations, soil total nitrogen (STN) concentrations and SOC:STN (mass ratio) in genetic soil horizons at Klosterhede. Lower-case letters within columns originate from pairwise comparison of horizon means. Means with no letter in common are significantly different (Tukey's HSD; $\alpha = 0.05$). Bold and bold-italic values indicate significant ($P < 0.05$) and marginally significant ($P < 0.1$) post-hoc differences between treatments within a horizon, respectively. Note that post-hoc differences were found for bulk density, STN in mineral horizons, and SOC:STN despite non-significant interactions. Means (±SE) were derived from 10-12 samples per horizon/treatment combination.

	Thickness (cm)	Soil mass ($kg\ m^{-2}$)	ρ_B ($g\ cm^{-3}$)	SOC ($mg\ g^{-1}$ soil)	STN ($mg\ g^{-1}$ soil)	SOC:STN (mass ratio)
Oe	*a*	*a*	*a*	*a*[†]	*a*[†]	*a*
Control	5.1 ± 0.4	4.9 ± 0.5	0.10 ± 0.01	500.3 ± 4.8	15.6 ± 0.6	32.6 ± 1.2
+ Nitrogen	5.5 ± 0.3	6.6 ± 0.6	0.12 ± 0.01	485.2 ± 4.3	15.5 ± 0.3	31.4 ± 0.7
Oa	*b*	*a*	*a*	*b*[†]	*b*[†]	*b*
Control	3.2 ± 0.6	3.8 ± 0.3	0.17 ± 0.03	***394.9 ± 9.2***	**10.2 ± 0.4**	39.2 ± 1.6
+ Nitrogen	3.4 ± 0.4	4.0 ± 0.5	0.13 ± 0.02	***413.3 ± 8.3***	**11.2 ± 0.4**	37.4 ± 1.2
AE	*c*	*b*	*b*	*a*[‡]	*a*[‡]	*c*
Control	**8.5 ± 0.5**	**76.5 ± 5.8**	**0.92 ± 0.03**	47.7 ± 3.5	***0.9 ± 0.1***	***56.9 ± 2.8***
+ Nitrogen	**6.5 ± 0.4**	**50.5 ± 4.1**	**0.81 ± 0.04**	53.9 ± 5.5	***1.1 ± 0.1***	***52.2 ± 2.7***
E	*cd*	*b*	*c*	*b*[‡]	*b*[‡]	*c*
Control	7.4 ± 0.6	***77.2 ± 7.2***	**1.07 ± 0.04**	27.4 ± 2.1	0.5 ± 0.0	50.6 ± 1.9
+ Nitrogen	6.9 ± 0.4	***64.2 ± 4.2***	**0.98 ± 0.03**	26.6 ± 1.6	0.5 ± 0.0	49.6 ± 1.8
Bh	*ad*	*c*	*b*	*a*[‡]	*c*[‡]	*b*
Control	5.1 ± 0.2	39.5 ± 2.2	0.81 ± 0.04	58.3 ± 2.6	1.5 ± 0.1	39.3 ± 0.9
+ Nitrogen	6.1 ± 0.5	47.4 ± 4.5	0.80 ± 0.04	56.6 ± 2.5	1.4 ± 0.1	41.1 ± 1.3
Bs[a]	*e*	*d*	*c*	*b*[‡]	*d*[‡]	*b*
Control	9.6 ± 1.2	104.8 ± 9.8	0.97 ± 0.04	29.4 ± 1.7	0.7 ± 0.1	42.0 ± 1.3
+ Nitrogen	10.6 ± 0.9	94.7 ± 4.5	0.96 ± 0.03	30.5 ± 1.2	0.8 ± 0.0	41.3 ± 1.6
Sign. effects	Horizon, *+N x Horizon*[b]	*+N*[c], Horizon, +N x Horizon	*+N*[d], Horizon	Org: Horizon, +N x Horizon Min: Horizon	Org: Horizon, +N x Horizon Min: Horizon	Horizon

[a] Lower horizon boundary of Bs horizons was normalized to 30 cm for calculation of horizon thickness. Soil mass and bulk density were calculated for original sampling depths of 31.6±1.3 cm and 29.7±0.8 cm from top of mineral soil in control and +N treatment plots, respectively. Marginally significant main effects and treatment x horizon interactions ([b]P=0.089,[c]P=0.085,[d]P=0.077).

[†] Lower-case letters indicate significant post-hoc differences between organic horizons.

[‡] Lower-case letters indicate significant post-hoc differences between mineral horizons.

Table S4.3 Summary of N-induced changes of direct controls of SOC and STN pools in Oe and A horizons. Black arrows indicate a significant N-induced increase/decrease compared to controls ($P < 0.05$). Grey arrows indicate non-significant trends.

	Alptal		Klosterhede	
	Oe horizon	Ah horizon	Oe horizon	AE horizon
Thickness	↑	↓	↑	↓
Bulk density	↑	↓	↑	↓
SOC concentration	↓	↓	↓	↑
STN concentration	↑	↓	↓	↑
SOC pool	↑	↓	↑	↓
STN pool	↑	↓	↑	↓

Appendix 5: Total belowground C and N pools – Supplementary Table

Table S5.1. Total belowground C and N pools at Alptal and Klosterhede. P-values are derived from linear mixed models including block and replicate within treatment and block as random effects (see Methods sections for details). In addition, bold and bold-italic values indicate significant ($P < 0.05$) and marginally significant ($P < 0.1$) differences between treatments, respectively. Means (±SE) were derived from 12 samples except for the control plots at Klosterhede (10 samples).

	Alptal		Klosterhede	
	C pool (kg m^{-2})	N pool (kg m^{-2})	C pool (kg m^{-2})	N pool (kg m^{-2})
Soil				
Control	11.91 ± 0.78	0.595 ± 0.033	14.23 ± 0.55	***0.332 ± 0.014***
+ Nitrogen	10.41 ± 0.79	0.526 ± 0.035	14.87 ± 0.36	***0.374 ± 0.014***
P	0.181	0.172	0.381	***0.055***
Fine roots (<2mm)				
Control	0.22 ± 0.04	0.004 ± 0.001	**0.21 ± 0.02**	**0.003 ± 0.000**
+ Nitrogen	0.24 ± 0.04	0.004 ± 0.001	**0.28 ± 0.03**	**0.006 ± 0.001**
P	0.974	0.884	**0.038**	**<0.001**
Soil and fine roots				
Control	12.13 ± 0.80	0.598 ± 0.034	14.43 ± 0.56	**0.335 ± 0.014**
+ Nitrogen	10.65 ± 0.80	0.530 ± 0.035	15.15 ± 0.36	**0.380 ± 0.014**
P	0.202	0.180	0.332	**0.044**

Appendix S6: Potential C mineralization rates and ecosystem-level C cycling

For the measurement of potential C mineralization rates, 5-20g of field-moist soil was evenly spread out over the bottom of cylindrical PVC-tubes (10 cm diameter), adjusted to 60% maximum water holding capacity and incubated at 10°C for three days approximating the mean annual temperature at our sites. During incubation, PVC-tubes were covered with Parafilm® to allow for gas exchange but prevent water loss. After incubation, respiration rates were measured at room temperature with a portable infrared gas analyzer (LI-8100A, LI-COR Bioscience, Lincoln, NE, U.S.) equipped with a 10-cm diameter survey chamber (8100-102, LI-COR Bioscience). For each measurement, the survey chamber was placed on top of the PVC-tube to create a closed-chamber system with a defined headspace volume (1134.8 cm^3). Then, CO_2 concentrations in the closed-chamber system were monitored over two minutes (pre-purge: 30s, post-purge: 10s) with a reading taken every second. Mixing and pressure equalization of chamber volume during measurements was ensured by the LI-COR system. Fluxes of CO_2 (µmol CO_2 m^{-2} s^{-1}) were obtained by fitting non-linear regression models to the CO_2 data using the manufacturers FV8100v3.1.1 software package. The resulting area-based fluxes were normalized to soil dry mass and SOC concentrations (µg CO_2-C g^{-1} soil h^{-1} and µg CO_2-C g^{-1} soil C h^{-1}) using the molar mass of C (12 g mol^{-1}), the area over which the soil was spread out (0.0078 m^2) and measurements of gravimetric water content and SOC concentrations.

Figure S6.1 Potential C mineralization rates per g soil (a,c) and per g SOC (b,d) in genetic soil horizons at Alptal (upper panels) and Klosterhede (lower panels). Light grey circles depict data from controls plots, dark gray triangles depict data from N addition plots. Lower-case letters next to the right border of each plate originate from pairwise comparison of horizon means. Means with no letter in common are significantly different (Tukey's HSD; $\alpha = 0.05$). Note that there was a significant post-hoc difference between treatments for specific respiration rates in the Oe horizon at Klosterhede (d, $P < 0.001$). Means (±SE) were derived from 4 samples per horizon/treatment combination.

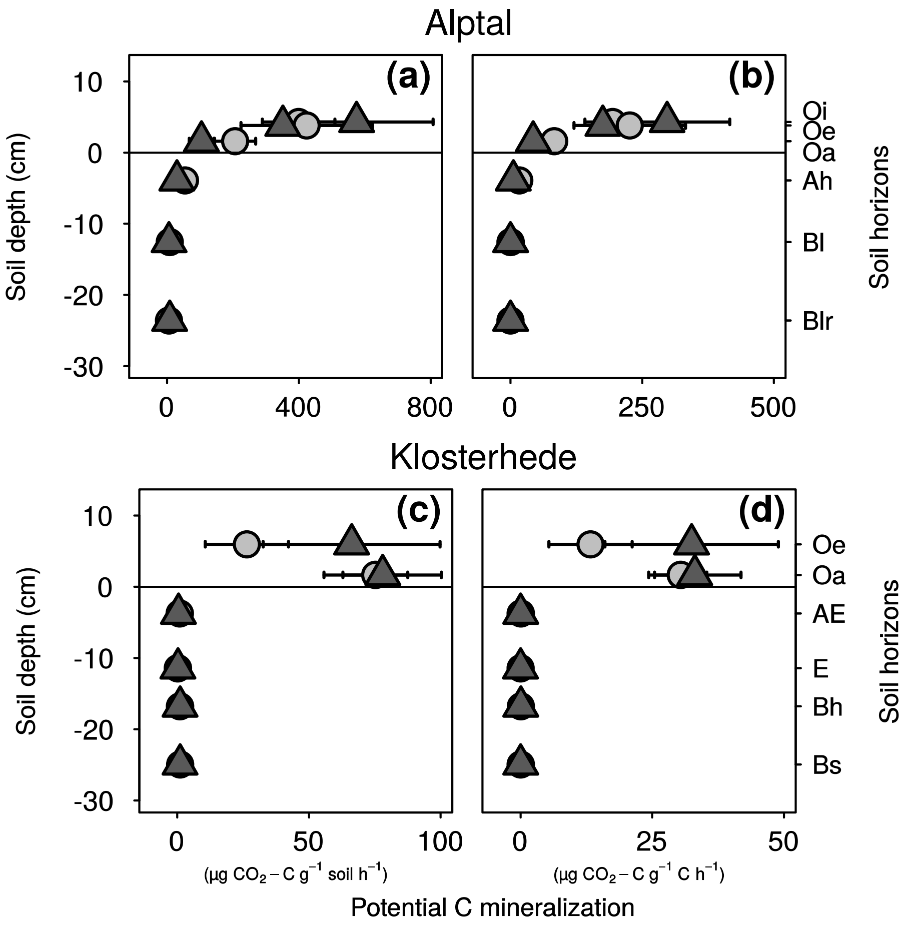

172 **Figure S6.2** Effects of N addition on selected C cycling parameters at Alptal (a) and Klosterhede (b). See Tables S6.1 and S6.2 for details.

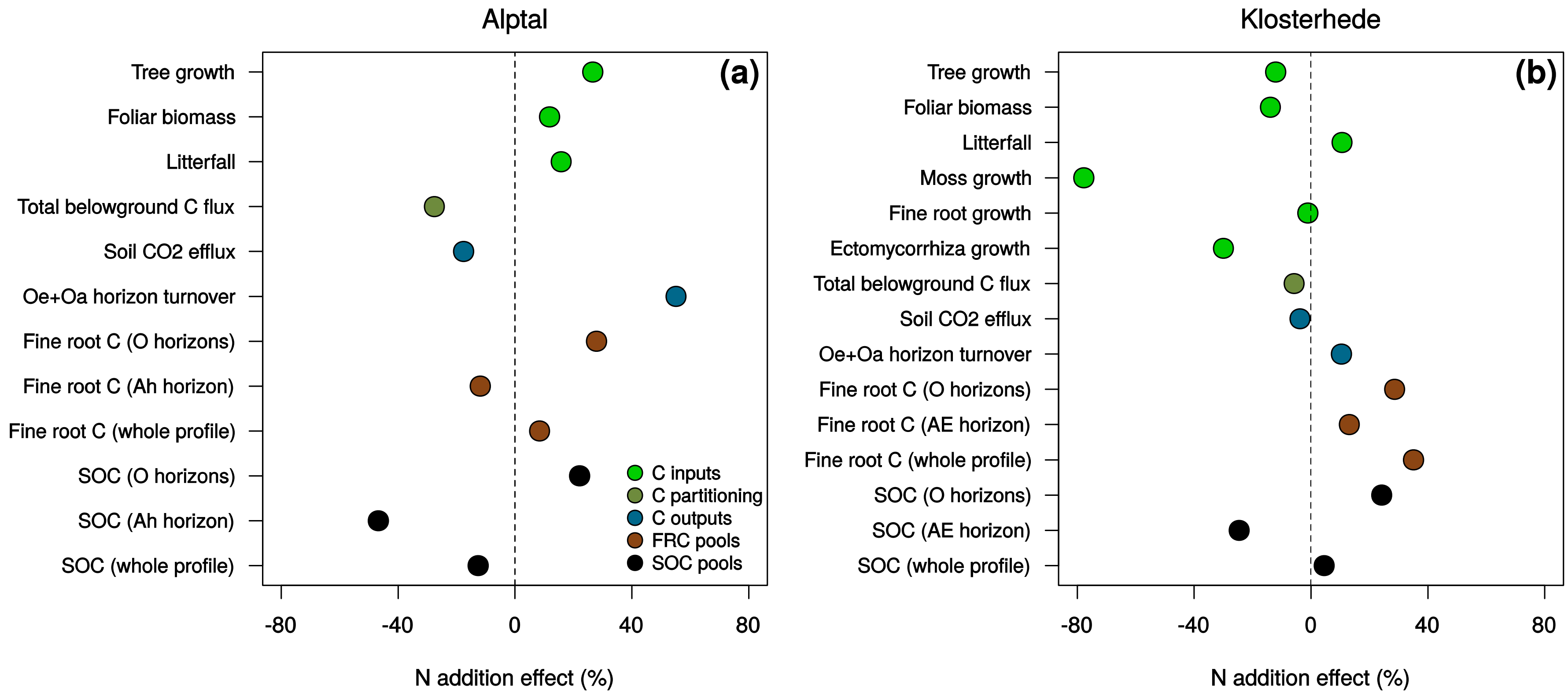

Table S6.1 Carbon inputs, C partitioning, C outputs and belowground C pools in response to long-term N addition at Alptal.

	Units	Control	+ Nitrogen	% Change	Level of significance	Time	Reference
C inputs							
Tree growth rates (Basal area increment)[A]	$mm^2\ y^{-1}$	632.0	800.5	27	n.a.	1960-2008	Krause and others 2012
Foliar biomass	mg $needle^{-1}$	5.5	6.1	12	n.a.	1995-2009	Krause and others 2012
Foliar litterfall[B]	g C $m^{-2}\ y^{-1}$	113.3	131.2	16	n.a.	1995-2008[C]	P. Schleppi unpublished results
C partitioning							
Total belowground C flux (TBCF)[D]	g C $m^{-2}\ y^{-1}$	300.0	217.1	-28	n.a.	1995-2012	This study
Belowground net primary production (BNPP)[E]	g C $m^{-2}\ y^{-1}$	126.0	91.2	-28	n.a.	1995-2012	This study
C outputs							
In-situ soil CO_2 efflux	g C $m^{-2}\ y^{-1}$	421.0	347.0	-18	n.a.	2007-2012	Krause and others 2013
Methane flux	g C $m^{-2}\ y^{-1}$	0.0	0.1	1100	n.a.	2007-2012	Krause and others 2013
Potential soil respiration – Oi	µg CO_2-C g^{-1} soil h^{-1}	399.3	575.2	44	n.s. (P= 0.718, Dunnett's test)	2014	This study
Potential soil respiration – Oe	µg CO_2-C g^{-1} soil h^{-1}	423.9	351.3	-17	n.s. (P= 0.589, Dunnett's test)	2014	This study
Potential soil respiration – Oa	µg CO_2-C g^{-1} soil h^{-1}	206.6	104.3	-50	n.s. (P= 0.221, Dunnett's test)	2014	This study
Potential soil respiration – Ah	µg CO_2-C g^{-1} soil h^{-1}	54.0	29.7	-45	n.s. (P= 0.430, Dunnett's test)	2014	This study
Potential soil respiration – Bl	µg CO_2-C g^{-1} soil h^{-1}	8.3	6.0	-28	n.s. (P= 0.603, Dunnett's test)	2014	This study
Potential soil respiration – Blr	µg CO_2-C g^{-1} soil h^{-1}	5.9	7.9	34	n.s. (P= 0.387, Dunnett's test)	2014	This study
Oe+Oa horizon turnover[F]	years	11.9	18.5	55	n.a.	1992-2014	This study
C pools							
Fine root C pool in Ohorizons	g C m^{-2}	61.1	78.2	28	n.s. (P= 0.978, Dunnett's test)	2014	This study
Fine root C pool in Ah horzion	g C m^{-2}	138.1	121.7	-12	n.s. (P= 0.588, Dunnett's test)	2014	This study
Fine root C pool in Bl horizon	g C m^{-2}	16.8	24.9	48	n.s. (P= 0.997, Dunnett's test)	2014	This study
Fine root C pool in Blr horizon	g C m^{-2}	2.9	12.8	335	n.s. (P= 0.200, Dunnett's test)	2014	This study
Fine root C pool in whole profile	g C m^{-2}	219.0	237.5	8	n.s. (P= 0.974, mixed model)	2014	This study
Soil C pool in O horizons	kg C m^{-2}	1.3	1.6	22	n.s. (P= 0.304, Dunnett's test)	2014	This study
Soil C pool in Ah horizon	kg C m^{-2}	4.4	2.4	-47	** (P= 0.002, Dunnett's test)	2014	This study
Soil C pool in Bl horizon	kg C m^{-2}	2.6	3.0	15	n.s. (P= 0.706, Dunnett's test)	2014	This study
Soil C pool in Blr horizon	kg C m^{-2}	3.2	3.3	2	n.s. (P= 0.752, Dunnett's test)	2014	This study
Soil C pool in whole profile	kg C m^{-2}	11.9	10.4	-13	n.s. (P= 0.181, mixed model)	2014	This study

[A] "Control" refers to the average annual BAI increments on the future N addition plots from 1960-1994 before N additions started, "+ Nitrogen" refers to BAI increments of N addition plots within the period of N additions, i.e. from 1995 to 2008.
[B] Assuming 50% of litter dry mass is C.
[C] Excluding data from 2003 where litterfall was unusually high in N addition plots due to a bark beetle infestation.
[D] Calculated as in-situ soil CO_2 efflux minus foliar litterfall (see Raich & Nadelhoffer 1989, Litton & Giardina 2008).
[E] Assuming that 0.42 of TBCF is allocated to BNPP at MAT=6°C (Litton & Giardina 2008).
[F] Calculated as Oe+Oa mass divided by foliar litterfall (see Zak et al. 2008).
n.a. = not analyzed
n.s. = not significant at P<0.05

Annual tree growth rates were calculated from basal area increments determined on tree rings (Krause and others 2012). Estimates of foliar biomass were derived from needle samples taken yearly from the seventh whorl from five tree tops per plot. For each of the five most recent needle age classes, 120 needles were dried to constant weight (65°C) before needle dry mass was determined (Schleppi and others 1999; Krause and others 2012). Foliar litterfall was sampled every second week from four collectors (0.12 m^2) per plot (Schleppi and others 1999; Krause and others 2013). Soil CO_2 efflux and CH_4-C fluxes were measured by repeated sampling of the headspace of closed PVC chambers (height: 30 cm, inner diameter: 32 cm) in each plot (Krause and others 2013).

182 **Table S6.2** Carbon inputs, C partitioning, C outputs and belowground C pools in response to long-term N addition at Klosterhede.

C inputs							
Tree growth rates (Basal area increment)	$m^2\ ha^{-1}\ y^{-1}$	0.865	0.761	-12	n.a.	1992-2014	Ginzburg (2014)
Tree growth rates (Basal area increment)	$m^2\ ha^{-1}\ y^{-1}$	0.895	0.680	-24	n.a.	2004-2014	Ginzburg (2014)
Foliar biomass	$mg\ needle^{-1}$	4.527	3.897	-14	n.a.	1992-2009	P. Gundersen unpublished results
Foliar litterfall[A]	$g\ C\ m^{-2}\ y^{-1}$	112.1	123.9	11	n.a.	1992-2010	Ginzburg (2014)
Moss growth	$g\ C\ m^{-2}\ y^{-1}$	9.0	2.0	-78	n.a.	2013	Ginzburg (2014)
Fine root growth	$g\ m^{-2}\ y^{-1}$	177	175	-1	n.s. (P=0.7, paired t-test)	2007-2008	Ginzburg (2014)
EM growth	$g\ m^{-2}\ y^{-1}$	10	7	-30	n.s. (P=0.3, t-test)	2010	Ginzburg (2014)
C partitioning							
Total belowground C flux (TBCF)[B]	$g\ C\ m^{-2}\ y^{-1}$	775.6	730.3	-6	n.a.	1992-2010	This study
Belowground net primary production (BNPP)[C]	$g\ C\ m^{-2}\ y^{-1}$	349.0	328.6	-6	n.a.	1992-2010	This study
C outputs							
In-situ soil CO_2 efflux	$mg\ CO_2\text{-}C\ m^{-2}\ h^{-1}$	101.3	97.5	-4	n.a.	2002-2003	Ginzburg (2014)
In-situ soil CO_2 efflux – interpolation[D]	$mg\ CO_2\text{-}C\ m^{-2}\ h^{-1}$	103.3	101.7	-2	n.a.	2002-2003	Ginzburg (2014)
Litter decomp.– exogenous N, 0-18 mon.	% accum. mass loss	23.2	20.7	-11	n.a.	2001-2006	Ginzburg (2014)
Litter decomp. – exogenous N, 24-60 m.	% accum. mass loss	60.8	58.2	-4	n.a.	2001-2006	Ginzburg (2014)
Litter decomp. – exogenous N, 0-18 m.	% mass loss $month^{-1}$	2.1	1.8	-13	n.a.	2001-2006	Ginzburg (2014)
Litter decomp. – exogenous N, 24-60 m.	% mass loss $month^{-1}$	1.1	1.2	9	n.a.	2001-2006	Ginzburg (2014)
Litter decomp. – endogenous N, 0-18 m.	% accum. mass loss	20.3	23.5	16	n.a.	2001-2006	Ginzburg (2014)
Litter decomp. – endogenous N, 24-60 m.	% accum. mass loss	60.3	58.7	-3	n.a.	2001-2006	Ginzburg (2014)
Litter decomp. – endogenous N, 0-18 m.	% mass loss $month^{-1}$	1.9	2.1	12	n.a.	2001-2006	Ginzburg (2014)
Litter decomp. – endogenous N, 24-60 m.	% mass loss $month^{-1}$	1.3	1.0	-23	n.a.	2001-2006	Ginzburg (2014)
Potential C mineralization – LF (Oie)	$mg\ CO_2\text{-}C\ g^{-1}$ soil C h^{-1}	0.031	0.040	29	n.s.	2003	Ginzburg (2014)
Potential C mineralization – H (Oa)	$mg\ CO_2\text{-}C\ g^{-1}$ soil C h^{-1}	0.020	0.017	-14	n.s.	2003	Ginzburg (2014)
Potential C mineralization – Min	$mg\ CO_2\text{-}C\ g^{-1}$ soil C h^{-1}	0.011	0.010	-6	n.s.	2003	Ginzburg (2014)
Potential C mineralization – LF (Oie)	$mg\ CO_2\text{-}C\ g^{-1}$ soil C h^{-1}	0.022	0.019	-15	n.s.	2013	Ginzburg (2014)
Potential C mineralization – H (Oa)	$mg\ CO_2\text{-}C\ g^{-1}$ soil C h^{-1}	0.010	0.009	-13	n.s.	2013	Ginzburg (2014)
Potential C mineralization – Min	$mg\ CO_2\text{-}C\ g^{-1}$ soil C h^{-1}	0.016	0.019	15	n.s.	2013	Ginzburg (2014)

[A] Assuming 50% of litter dry mass is C.
[B] Calculated as in-situ soil respiration (converted to $g\ C\ m^{-2}\ y^{-1}$) minus foliar litterfall (see Raich & Nadelhoffer 1989, Litton & Giardina 2008).
[C] Assuming that 0.45 of TBCF is allocated to BNPP at MAT=10°C (Litton & Giardina 2008).
[D] In-situ soil respiration was measured on different occasions during 2002 (April, June, Oktober, November, December) and 2003 (February, June, August). In addition to reporting average values from these measurements, we linearly interpolated fluxes between measurements and calculated average fluxes from this interpolated dataset.

Table S6.2 – continued

	Units	Control	+ Nitrogen	% Change	Level of significance	Time	Reference
C outputs – continued							
Potential C mineralization – Oe	µg CO_2-C g^{-1} soil h^{-1}	26.5	66.2	150	n.s. (P=0.140, Dunnett's test)	2014	This study
Potential C mineralization – Oa	µg CO_2-C g^{-1} soil h^{-1}	75.2	78.0	4	n.s. (P=0.907, Dunnett's test)	2014	This study
Potential C mineralization – AE	µg CO_2-C g^{-1} soil h^{-1}	0.97	0.51	-47	n.s. (P=0.170, Dunnett's test)	2014	This study
Potential C mineralization – E	µg CO_2-C g^{-1} soil h^{-1}	0.25	0.28	12	n.s. (P=0.912, Dunnett's test)	2014	This study
Potential C mineralization – Bh	µg CO_2-C g^{-1} soil h^{-1}	1.06	1.10	4	n.s. (P=0.937, Dunnett's test)	2014	This study
Potential C mineralization – Bs	µg CO_2-C g^{-1} soil h^{-1}	1.14	1.10	-4	n.s. (P=0.720, Dunnett's test)	2014	This study
Oe+Oa horizon turnover[E]	years	38.5	42.5	10	n.a.	1992-2014	This study
C pools							
Moss C pool	g C m^{-2}	72.0	15.0	-79	n.a.	2013	Ginzburg (2014)
Fine root C pool in O horizons	g C m^{-2}	89.0	114.5	29	n.s. (P=0.123, Dunnett's test)	2014	This study
Fine root C pool in AE horzion	g C m^{-2}	43.6	49.3	13	n.s. (P=0.624, Dunnett's test)	2014	This study
Fine root C pool in E horizon	g C m^{-2}	24.0	30.3	26	n.s. (P=0.294, Dunnett's test)	2014	This study
Fine root C pool in Bh horizon	g C m^{-2}	23.4	26.6	14	n.s. (P=0.475, Dunnett's test)	2014	This study
Fine root C pool in Bs horizon	g C m^{-2}	39.3	59.1	50	n.s. (P=0.258, Dunnett's test)	2014	This study
Fine root C pool in whole profile	g C m^{-2}	207.1	279.8	35	* (P=0.038, mixed model)	2014	This study
Soil C pool in O horizons	kg C m^{-2}	3.9	4.9	24	* (P=0.037, Dunnett's test)	2014	This study
Soil C pool in AE horizon	kg C m^{-2}	3.5	2.6	-25	* (P=0.024, Dunnett's test)	2014	This study
Soil C pool in E horizon	kg C m^{-2}	2.2	1.7	-24	n.s. (P=0.191 Dunnett's test)	2014	This study
Soil C pool in Bh horizon	kg C m^{-2}	2.3	2.7	19	n.s. (P=0.357, Dunnett's test)	2014	This study
Soil C pool in Bs horizon	kg C m^{-2}	2.6	2.9	13	n.s. (P=0.286, Dunnett's test)	2014	This study
Soil C pool in whole profile	kg C m^{-2}	14.2	14.9	5	n.s. (P=0.381, mixed model)	2014	This study

[E] Calculated as Oe+Oa mass divided by foliar litterfall (see Zak et al. 2008).
n.a. = not analyzed
n.s. = not significant at P<0.05

Tree growth rates were calculated from changes in basal area increment (BAI), which were derived from annual measurements of tree diameter at breast height (DBH) since 1990 (Ginzburg 2014). Estimated of foliar biomass was derived from 100 current year needles taken from the seventh whorl of 5 randomly selected trees per plot and year (P. Gundersen, pers.communication). Litterfall was collected monthly

in 10 traps per plot in the years 1991-1995, 2002 and 2010. The foliar fraction was sorted out, dried at 55°C to constant mass and weighted (Ginzburg 2014).

Moss growth was estimated by a three step-procedure: First, moss cover (%) of control and N addition plots was visually estimated in 1991-1995 and in 2013. Then, moss cover was converted into moss biomass using the cover-biomass relationships measured in three fully covered 10 cm x 10 cm squares taken from the control plots. Finally, moss biomass was divided by an assumed lifetime of 8 years to calculated annual moss growth (Ginzburg 2014).

Fine root growth was estimated during 2007-2008 using ingrowth cores (Ginzburg 2014)7). Growth of ectomycorrhizal fungi was estimated in 2009-2010 using ingrowth bags (mesh sizes 50 µm; (Ginzburg 2014). In-situ soil C efflux rates were measured during 2002 in 15 PVC collars per treatment with continous-flow chamber attatched to an infrared gas analyzer (Ginzburg 2014).

Litter decomposition was measured after 10 years of N addition treatments in a reciprocal transplant experiment to separate the effects of N on litter traits (endogenous N effect) and soil environment (exogenous N effect). Therefore, dried needle litter from control and N addition plots was place in nylon mesh litterbags (0.5 mm x 1mm mesh size) and a total of 360 litterbags were deployed in control and N addition plots in 2001. Sets of 5 replicated litterbags were collected after 6, 12, 18, 24, 32 and 60 month from 3 random locations within control and N addition plots. Replicates were pooled to 3 composite samples per treatment before dry mass was determined (Ginzburg 2014). Potential C mineralization rates were measured in 2003 and 2013 on six and four replicates per treatment and soil layer (LF, H, Min) using a Respirometer (Ginzburg 2014).

Appendix S7: Statistical considerations regarding the experimental layout at Klosterhede

The original experimental layout at Klosterhede posed some challenges before the experiment could be analyzed as a split block design. Originally, the experiment included one single area over which N has been applied, framed by two control areas (see Figure S7.1). As such, there are strictly speaking just two plots at Klosterhede, one which received N and one that did not, even though the control plot consists of two areas which are located on two of the sides of the N addition plot. If we had analyzed the experiment as suggested by the original spatial arrangement of plots, we would have been been able to use inferential statistics to test for differences between treatments at that special location, but inference beyond the experimental site would not have been valid. However, the main aim of our study was to learn (infer) from this unique long-term experiment how decadal N addition affects C pools in temperate forests generally, and we feel that our reinterpretation of the original study design allows us to do so.

Thus, we first divided the original N addition and control plots into four subplots each and then blocked one N subplot with an adjacent control subplot (see Figure S7.1). This was not done to uncritically increase replication, but to *post-hoc* mimick a blocked design, similar to that employed at Alptal. In this view, each block at Klosterhede contains two experimental units (henceforth termed "plots") to which treatments have been applied. The resulting analytical approach is similar to a situation in which the treatments would have been applied at a block level in the first place. Hence, we employed linear (mixed) models to asses effects of horizon and N addition treatment at Klosterhede.

A main caveat remains: Treatments have not been assigned randomly to plots within blocks. However, we stress that it is not the lack of random assignment *per se* that is of concern as "randomization is simply a way of achieving interspersion in a way that eliminates the possibility of bias [...]" (Hurlbert 1984, 192). Even strict randomization would have produced the employed layout with a probability of 6.25%, assuming a 50% chance for each plot to be assigned to one of the two treatments within a block.

Rather, it is the lack of interspersion/the spatial segregation of treatments that can lead to a lack of independence of errors, thus would have restricted our ability to exactly know if the probability of type I errors were 5% as assumed in the analysis, and precluded inference (Hurlbert 1984, 192). This is because interspersion of treatments controls for (1) initial differences or gradients between plots within a block and (2) for differences between plots that either arise or intensify during the experiment as a result of non-demonic intrusion (Hurlbert 1984, 195).

For lack of interspersion not to invalidate our analysis, we have to show that (1) no within-block gradients existed before treatments were applied and (2) that any non-demonic intrusion did not selectively affect the spatially segregated N addition treatments so that no within-block gradients arose during the experiment. We believe that strong gradients did not exist before the start of the experiment for the following reasons:

(1) The parent material at Klosterhede has been judged to be a “homogenous sandy, nutrient poor glacio-fluvial outwash plain” (Gundersen and Rasmussen 1995). This indicates that strong gradients in soil properties due to variations in parent material are unlikely.

(2) The site has been ploughed prior to forest establishment. Thus it is reasonable to assume a high degree of homogeneity in initial soil properties compared to sites where natural soil development progressed unaltered.

We further employed two unconstrained ordination techniques (DCA, PCA) to evaluate if strong gradients were present across the experimental area (and thus within blocks) at the time of sampling, either as a consequence of pre-existing gradients or due to “non-demonic intrusion” during the experiment. Specifically, we tested if

(Objective 1) multivariate gradients were detectable across the experimental area in variables not significantly affected by N addition treatment (mineral soil texture, EOC, mineral soil

CEC). For this analysis, we used data from mineral horizons of all eight plots, where texture and CEC were measured.

(Objective 2) multivariate gradients were detectable in variables significantly affected by N addition treatment in at least one horizon (thickness, bulk density, %C, NH_4-N, NO_3-N, EON). Here, we only used data from the four control plots. The spatial arrangement of the control plots (see Figure S6.1) allowed us to check for gradients independent of N addition across the experimental area at the time of sampling.

First, we used detrended correspondence analysis (DCA) to investigate the length of possible gradients within each horizon. The length of DCA axes indicates the length of ecological gradients (Hill and Gauch 1980) which can be interpreted as the "beta diversity in community composition (the extent of species turnover) along the individual independent gradients [...]" (Lepš and Šmilauer 2003, 51) in case the response is multivariate species abundance. However, the reasoning also applies to any response for which unimodal distribution is assumed. If the DCA axis is smaller than 3 SD units, the ecological gradient is regarded as "short". If it is larger than 4 SD units, the "turnover" in the multivariate response is high, the gradient is regarded as "long"(Lepš and Šmilauer 2003).

Second, we conducted two PCAs for each soil horizon. The first PCA was based on soil texture, EOC and CEC and was carried out for mineral horizons of all eight plots (Objective 1). The second PCA was based on thickness, bulk density, %C, NH_4-N, NO_3-N and EON and was carried our for all horizons of the four control plots (Objective 2).

With regard to Objective 1, we found only short within-horizon gradients across all eigth plots as indicated by the lengths of the first DCA axes (<0.6 SD). Horizon-specific PCAs further revealed that the first three axes accounted for ~90% of the variance within each horizon, and that plots generally did not separate along PCA axes. The only exception was found within the AE horizon where plots of

block D were significantly separated along PC3 (11.5% of total variance). This was driven by a higher CEC in this block.

With regard to Objective 2, DCA also revealed short gradients (<0.7 SD). Within this relatively low spatial variation, some control plots were separated along PCA axes when the variables affected by N addition were considered. These separations, however, were horizon- and block-specific: In the Oe horizon, the control plot of block C separated along PC1 (48.3% of total variance) which was driven by on average higher amounts of NH_4-N and EON as well as a smaller horizon thickness. The AE horizon of the control plot in block A was relatively thick on average, which drove separation along PC2 (58.1% of total variance). Further, the Bh horizon of block B had a lower bulk density on average and therefore was separated along PC2 in the respective PCA (52.9% of total variance).

In summary, we did not find strong gradients across plots in variables unaffected by N addition (Objective 1). Similarly, we did not find strong, consistent within-horizon gradients between control plots (Objective 2). Still, slight differences between blocks indicate that some caution is required in interpreting the effect of N addition on horizon thickness and derived quantities. We conclude that slight differences in horizon thickness unrelated to N addition might have been present at Klosterhede, but do not affect our general conclusions, i.e. that N addition resulted in an vertical redistribution of SOC pools from AE to Oe horizons.

Figure S7.1 Sketch of the experimental set-up at Klosterhede modified from Gundersen and Rasmussen (1995). The layout consists of one area over which N has been applied, framed by two control areas. Nitrogen additions started in February 1992. Quadrants sharing the same uppercase letter were treated as block in the statistical analyses.

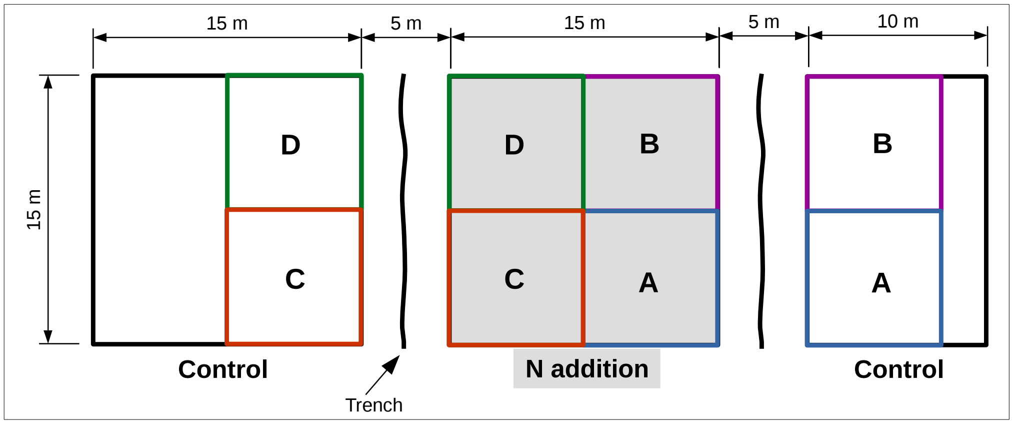

Supplementary References

Birouste M, Zamora-Ledezma E, Bossard C, Pérez-Ramos IM, Roumet C. 2014. Measurement of fine root tissue density: a comparison of three methods reveals the potential of root dry matter content. Plant Soil 374:299–313.

Ginzburg SO. 2014. Nitrogen Deposition Effects on Soil Carbon Dynamics in Temperate Forests. Unpublished Doctoral Thesis. University of Copenhagen, Denmark.

Gundersen P, Rasmussen L. 1995. Nitrogen mobility in a nitrogen limited forest at Klosterhede, Denmark, examined by NH4NO3 addition. For Ecol Manag 71:75–88.

Hurlbert SH. 1984. Pseudoreplication and the Design of Ecological Field Experiments. Ecol Monogr 54:187–211.

Krause K, Cherubini P, Bugmann H, Schleppi P. 2012. Growth enhancement of Picea abies trees under long-term, low-dose N addition is due to morphological more than to physiological changes. Tree Physiol 32:1471–81.

Krause K, Niklaus PA, Schleppi P. 2013. Soil-atmosphere fluxes of the greenhouse gases CO2, CH4 and N2O in a mountain spruce forest subjected to long-term N addition and to tree girdling. Agric For Meteorol 181:61–8.

Lepš J, Šmilauer P. 2003. Multivariate Analysis of Ecological Data using CANOCO. Cambridge University Press

Schleppi P, Muller N, Edwards PJ, Bucher JB. 1999. Three years of increased nitrogen deposition do not affect the vegetation of a montane forest ecosystem. Phyton 39:197–204.
